# Study on optimization algorithm of tuned mass damper parameters to reduce vehicle-bridge coupled vibration

**DOI:** 10.1371/journal.pone.0215773

**Published:** 2019-04-23

**Authors:** Jianwei Liu, Dejian Li, Peng Yu

**Affiliations:** 1 School of Civil Engineering, Central South University, Changsha, Hunan, China; 2 Changsha Planning & Design Institute Co., Ltd., Changsha, Hunan, China; University of New South Wales, AUSTRALIA

## Abstract

A vehicle-bridge tuned mass damper (TMD) coupled dynamic analysis and vibration-control model was established to optimize TMD damping effects on a steel-box girder bridge bearing vehicle loads. It was also used to investigate optimization efficiency of different algorithms in TMD design parameters. This model simulated bridges and vehicles with the use of a 7 degrees of freedom curved-beam element model and a 7 degrees of freedom vehicle model, respectively. The TMD system was simulated with the use of multiple rigid-body systems linked with springs and dampers. Road surface condition, as a vibration source, was simulated with the use of a frequency equivalent method based on a power spectrum. A variably-accelerated pattern search algorithm was proposed in line with the initial TMD parameters calculated by Den Hartog formula. Visual software was compiled by Fortran and used for an optimization study of vibration reduction. A three-span, curved, continuous steel-box girder bridge was used as the numerical example. Optimized effects and computational efficiency of vibration reduction under different methods were compared. The comparison included a single variable optimization based on Den Hartog formula, an ergodic search method, an integer programming method, a traditional genetic algorithm, a traditional pattern search algorithm, and a variably-accelerated pattern search algorithm. The results indicate that variably-accelerated pattern search algorithm is more efficient at improving TMD optimal parameter design. Final TMD parameter optimization values obtained by different methods are quite close to each other and tends verify the reliability of the optimization results.

## Introduction

Vehicles moving on irregular bridge road surfaces produce coupling vibrations in the vehicle-bridge system. Large amplitude causes noise and driving discomfort and generates fatigue damage to the bridge structure. Presently steel-box girder bridges are widely used as urban overpasses as they can be rapidly constructed and do not need full construction support. Vibration and noise remain as drawbacks. Vibration control research on steel-box girder bridge dynamic responses under vehicle loads is of great theoretical significance and practical engineering value. Prior studies tended to analyze the coupled dynamic response of vehicle-bridge system [1–5]. Recent studies [6–8] suggested that studies of coupled-systems vibration control should be included and that system damping could be achieved through control techniques such as tuned mass damper (TMD). Kwon et al., in their study of TMD damping and optimization, analyzed TMD vibration control effects on a three-span continuous girder bridge simulated with six degrees of freedom (DOF) beam element [9]. Guo and Lu [10] concluded that a Den Hartog (DH) optimization formula [11] could be applied to vibration control using TMD on a simple-supported beam bridge for high-speed trains. Miguel et al. proposed a novel hybrid stochastic-deterministic algorithm for optimal multiple tuned mass dampers (MTMD) design under seismic excitation [12]. Tubino and Piccardo proposed a numerical optimization criterion based on an efficiency factor maximization defined as "the ratio between the uncontrolled acceleration standard deviation and the controlled one to optimize TMD parameters and mitigate human-induced vibrations of pedestrian bridges" [13]. Fan et al. solved MTMD optimal parameters under arbitrary distribution of damping ratio and frequency ratio using a genetic algorithm (GA) [14]. Li et al. established a power balance equation for a structure-TMD system and obtained a power consumption formula for the main structure when the structural substrate was subjected to harmonic load [15]. They obtained TMD optimal frequency ratios and damping ratios by establishing an optimal goal of minimizing main structure energy consumption and comparing it to the four analytical optimization methods proposed by Den Hartog [11], Warburton [16], Tsai and Lin [17], and Sadek et al. [18].

The present state of bridge damping research is focused on vibration control in railway concrete girder bridges and footbridges and optimizing TMD parameters based on a single variable analysis. Research on the unconstrained optimized damping of the TMD system of steel-box girder bridges under the effects of automobiles remains scarce. Vigorous restrictions have often been imposed to simplify calculations such as equal quality and even frequency distributions. A relatively simple linear search technology to obtain optimal TMD parameters is an example [13–15, 19, 20]. The results obtained in these studies are not necessarily close to an actual optimal answer, especially for systems having certain levels of randomness and complexity such as controlling vehicle-bridge coupling vibrations, which may produce a large differences from the actual optimal answer. Fan et al. [14] and Li et al. [15] used a GA algorithm to study TMD damping optimization in simple structures. Computational efficiency and research goal differences suggest that it may not necessarily apply to a vibration damping optimization analysis to vehicle-bridge coupling systems. This paper proposes a new variably-accelerated pattern search algorithm (VAPS) based on an improved initial value. This realizes vehicle-bridge coupling damping optimization for an unrestricted TMD system. A three-span, curved, continuous steel-box girder bridge was taken as the model for studying damping optimization of the vehicle-bridge coupled dynamic response using self-programmed software VBTS-1. Optimal TMD system parameters were obtained by comparing the damping optimization effects and the calculation efficiency of different optimization algorithms. TMD vibration damping optimization has a limited number of calculations. The VAPS algorithm addresses the difficulty of current solutions which are unable address calculation efficiencies and vibration damping effects. The work offers a possibility of dealing with the unconstrained optimization problems of large-scale MTMD systems. A post-optimization TMD prolongs bridge life spans and contributes to the sustainable development of urban infrastructure construction. This optimization algorithm provides a reference value in the the vibration reduction design of bridges and other structures.

## Vehicle-bridge-TMD system dynamics model

### 2.1. Bridge dynamics model

**Fig 1:**
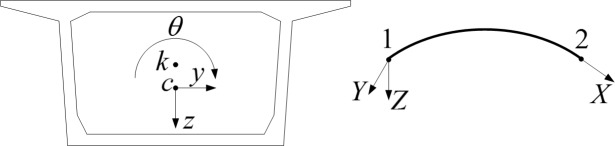
Beam element considering constraint torsion. *k* is the torsion center. *c* is mass center. *X* is bridge center curve coordinate calculated according to the unit arc length. *Y* is the radial coordinate. *Z* is the vertical coordinate.

The 7-DOF, spatial, curved-beam element, which takes constrained torsion into account, was used for the bridge in Fig 1.

Element displacement parameters are:

$$\{{\delta \}}_{e}={\{\begin{array}{c} \delta_{1}\\ \delta_{2} \end{array}\}}_{14\times 1}={\left[\begin{array}{ccccccc} x_{1} & y_{1} & z_{1} & \theta_{x1} & \theta_{y1} & \theta_{z1} & \varphi_{1}\\ x_{2} & y_{2} & z_{2} & \theta_{x2} & \theta_{y2} & \theta_{z2} & \varphi_{2} \end{array}\right]}^{T}$$ (1)

Subscript "1" and "2" are the left and right nodes respectively. *x*, *y*, *z*, and *θ* are linear and angular displacements in the *X*, *Y*, and *Z* directions. *φ* is the rate of change of X-axis direction torsional angle.

Rayleigh damping is adopted for the bridge model. The element characteristic matrix is formed according to the total potential energy invariance of the elastic system dynamics principle. The "check mark" rule is used to form the matrix [21]. Overall mass, damping, and stiffness matrix are obtained via coordinate transformation and assembly to establish a bridge dynamic equation:

$$M_{s}{\ddot{V}}_{s}+C_{s}{\dot{V}}_{s}+K_{s}V_{s}=P_{s}$$ (2)

In this formula (2), ***M***_*s*_,***C***_*s*_,***K***_*s*_ are structure mass, damping, and stiffness matrix in the global coordinate system, respectively. $V_{s},{\dot{V}}_{s},{\ddot{V}}_{s}$ are the displacement, velocity, and acceleration column vectors of the respective DOFs for the overall coordinates. ***P***_*s*_ is the structure external load vectors.

### 2.2. Vehicle dynamics model

**Fig 2:**
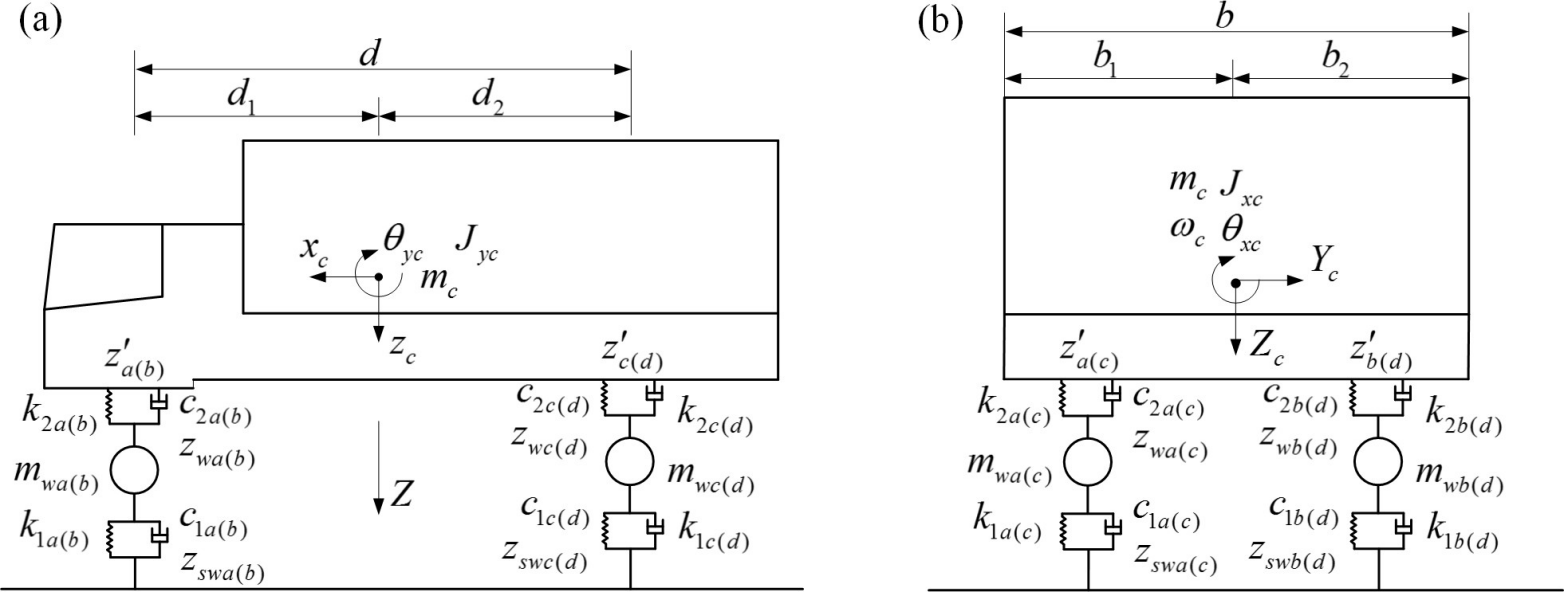
Simplified vehicle calculation model. (a) This is vehicle model side view. (b) This is vehicle model front view.

The vehicle is a 3-dimensional, 2-axis model consisting of 4 wheels and 1 body. A simplified model is in Fig 2. The model takes into account vertical flotation of the body and wheels (*z*_*c*_,*z*_*wa*_,*z*_*wb*_,*z*_*wc*_,*z*_*wd*_), front and rear body nodding (*θ*_*yc*_), and vehicle lateral rolling motion (*θ*_*xc*_) for a total of 7 DOFs. This fully simulates vehicle movement characteristics. Assuming that the vehicle is traveling at a constant speed parallel to the lane centerline, small displacement vibrations in the direction of the respective DOFs are considered. A centrifugal force for each concentrated mass acts on the respective mass center [22]. According to the principle of potential energy invariance and the "check mark" rule, a vehicle dynamics equation is formed (3). For detail meanings, see S1 Appendix and [21].

$$\mathrm{where}\;\begin{array}{l} M_{v}{\ddot{V}}_{v}+C_{v}{\dot{V}}_{v}+K_{v}V_{v}=P_{v}\\ \left\{ \begin{array}{l} M_{v}=\left[\begin{array}{cc} 0 & 0\\ 0 & M_{v} \end{array}\right]\\ C_{v}=\left[\begin{array}{cc} C_{sf}^{v} & C_{svo}\\ C_{vso} & C_{v}+C_{vf} \end{array}\right]\\ K_{v}=\left[\begin{array}{cc} K_{sf}^{v} & K_{svo}\\ K_{vso} & K_{v}+K_{vf} \end{array}\right]\\ V_{v}=\left[\begin{array}{c} V_{s}\\ V_{v} \end{array}\right],{\dot{V}}_{v}=\left[\begin{array}{c} {\dot{V}}_{s}\\ {\dot{V}}_{v} \end{array}\right],{\ddot{V}}_{v}=\left[\begin{array}{c} {\ddot{V}}_{s}\\ {\ddot{V}}_{v} \end{array}\right]\\ P_{v}=\left\{\begin{array}{c} p_{v}+p_{vscr}+p_{vskr}\\ p_{vvcr}+p_{vvkr} \end{array}\right\} \end{array}\right. \end{array}$$ (3)

### 2.3. Road surface condition

Studies indicate that road surface condition is a major factor affecting vehicle-bridge dynamic response [1, 23]. This paper constructs a frequency spectrum based on an equivalence method of the power spectrum frequency [24]. An irregular spectral density function is given in the Chinese national standard in GB/T 7031–2005 road vibration spectrum measurement data report. It is sampled discretely to construct a frequency spectrum.

The time domain sample transformed by Fourier is as (4):

$$x\left(n\right)=\frac{1}{N}\sum_{k=0}^{N-1}X\left(k\right)\exp\left(i\frac{2\pi kn}{N}\right),\left(n=0,1,\cdots ,N-1\right)$$ (4)

In Eq (4), *x*(*n*) is the excitation function that simulates road surface irregularity under the time domain. *X*(*k*) is the spectrum obtained by discretely sampling. *i* is the imaginary unit.

**Fig 3:**
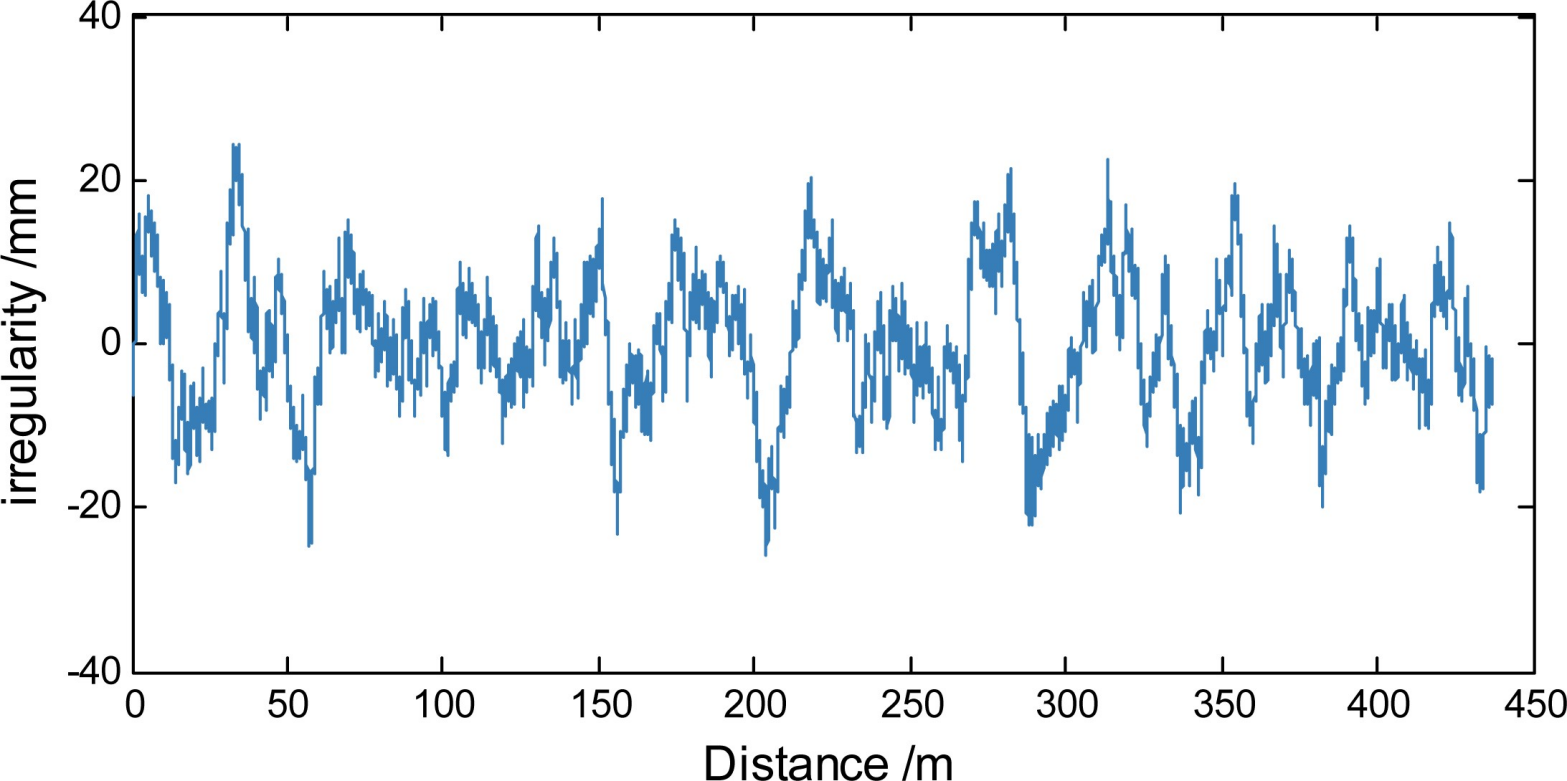
C-level road irregularity sample.

Numerical simulations for differing road irregularity grades are performed using this method. C-level irregularities are selected (Fig 3).

### 2.4. Vehicle-bridge-TMD system dynamic equation

**Fig 4:**
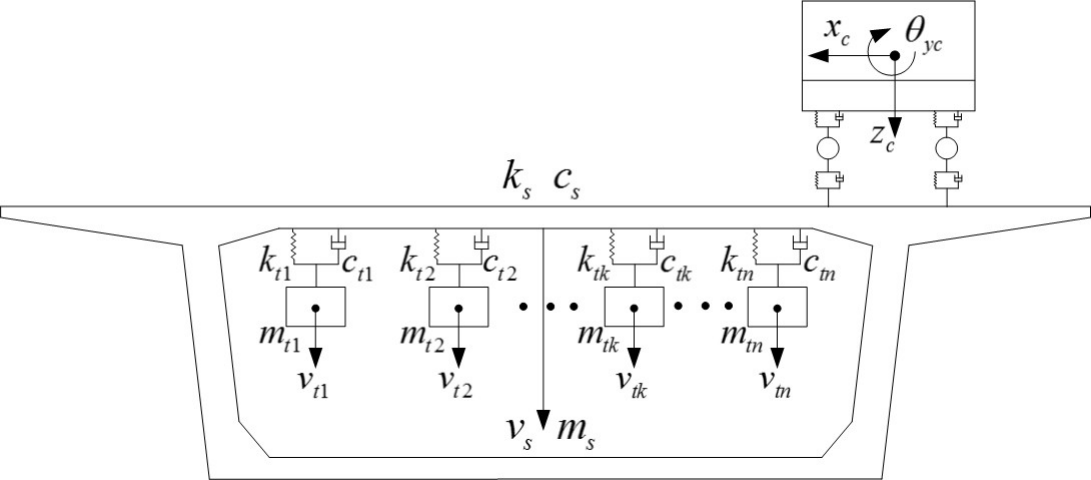
Vehicle-bridge-TMD coupled system dynamic model.

A single, tuned-mass damper (1-TMD) is simply represented as a spring, a mass, and a damping device. In practice, a larger 1-TMD is often designed as an *n*-TMD system composed of multiple small-mass TMDs. Every small TMD has the same frequency, and damping, ratios. This is called a distributed *n*-TMD system [14] as shown in Fig 4.

Given that excitation *p*(*t*) acts on the structure, the *k*th TMD dynamic equilibrium equation according to the d' Alembert principle is:

$$m_{tk}{\ddot{v}}_{tk}+c_{tk}\left({\dot{v}}_{tk}-{\dot{v}}_{s}\right)+k_{tk}\left(v_{tk}-v_{s}\right)=0\;(k=1,2,\cdots ,n)$$ (5)

The controlled, main structure, power balance equation is:

$$m_{s}{\ddot{v}}_{s}+c_{s}{\dot{v}}_{s}+k_{s}v_{s}-\sum_{k=1}^{n}c_{tk}\left({\dot{v}}_{tk}-{\dot{v}}_{s}\right)-\sum_{k=1}^{n}k_{tk}\left(v_{tk}-v_{s}\right)=p(t)$$ (6)

In Eq (6), *v*_*s*_ and *v*_*tk*_, are, respectively, vertical displacement of the main structure and the *k*th TMD relative to reference system. {*m*_*s*_,*c*_*s*_,*k*_*s*_} are the main structure’s mass, damping, and stiffness coefficients. {*m*_*tk*_,*c*_*tk*_,*k*_*tk*_} are the the *k*th TMD’s.

Based on bridge (5) and vehicle (6) dynamic equations, the bridge static equilibrium state is used as the vehicle-bridge system initial state. The external load vector ***P***_*s*_ = 0, is substituted into Eq (2). The matrices of bridges, vehicles, and TMDs are assembled to form a vehicle-bridge-TMD system spatial dynamic equation as follows:

$$\mathrm{where}\;\begin{array}{l} M_{vst}{\ddot{V}}_{vst}+C_{vst}{\dot{V}}_{vst}+K_{vst}V_{vst}=P_{vst}\\ \left\{ \begin{array}{l} M_{vst}=\left[\begin{array}{ccc} M_{s} & 0 & 0\\ 0 & M_{v} & 0\\ 0 & 0 & M_{t} \end{array}\right]\\ C_{vst}=\left[\begin{array}{ccc} C_{s}+C_{sf}^{v}+C_{sf}^{t} & C_{svo} & C_{sto}\\ C_{vso} & C_{v}+C_{vf} & 0\\ C_{tso} & 0 & C_{t} \end{array}\right]\\ K_{vst}=\left[\begin{array}{ccc} K_{s}+K_{sf}^{v}+K_{sf}^{t} & K_{svo} & K_{sto}\\ K_{vso} & K_{v}+K_{vf} & 0\\ K_{tso} & 0 & K_{t} \end{array}\right]\\ V_{vst}=\left[\begin{array}{c} V_{s}\\ V_{v}\\ V_{t} \end{array}\right],{\dot{V}}_{vst}=\left[\begin{array}{c} {\dot{V}}_{s}\\ \begin{array}{l} {\dot{V}}_{v}\\ {\dot{V}}_{t} \end{array} \end{array}\right],{\ddot{V}}_{vst}=\left[\begin{array}{c} {\dot{V}}_{s}\\ \begin{array}{l} {\dot{V}}_{v}\\ {\dot{V}}_{t} \end{array} \end{array}\right]\\ P_{vst}=\left[\begin{array}{c} p_{v}+p_{svcr}+p_{svkr}\\ p_{vvcr}+p_{vvkr}\\ 0 \end{array}\right] \end{array}\right. \end{array}$$ (7)

In Eq (7), the TMD system mass matrix is ***M***_*t*_ = *diag*(*m*_1_,*m*_2_,…,*m*_*n*_). The damping matrix is ***C***_*t*_ = *diag*(*c*_1_,*c*_2_,…,*c*_*n*_). The stiffness matrix is ***K***_*t*_ = *diag*(*k*_1_,*k*_2_,…,*k*_*n*_). The displacement column vector is ***V***_*t*_ = [*v*_1_,*v*_2_,…,*v*_*n*_]^T^. $C_{sf}^{t}=\sum_{k=1}^{n}c_{k}$, $K_{sf}^{t}=\sum_{k=1}^{n}k_{k}$, $C_{sto}=C_{tso}^{T}=-\left[c_{1},c_{2},\ldots ,c_{n}\right]$, $K_{sto}=K_{tso}^{T}=-\left[k_{1},k_{2},\ldots ,k_{n}\right]$ and others, provide detail meanings of each equation, see S1 Appendix and An et al. [21]. When the parameters ***M***_*t*_, ***K***_*t*_, and ***C***_*t*_ are the same, it is the distributed TMD system.

## Traditional numerical optimization methods of TMD system parameters

Steel structure low-frequency vibration takes displacement-based fatigue strength failure into account [25]. A TMD system design parameter optimization goal (mass ratio *μ*, frequency ratio *α*, and damping ratio *ζ*) is to find a suitable combination of parameters {*μ*_opt_,*α*_opt_,*ζ*_opt_} and to minimize the bridge displacement response *D*_*z*_ under the vehicle load:

$$\underset{\mu ,\alpha ,\xi }{\min}\left(\underset{p(t)}{\max}\;{}^{D_{z}}\right)$$ (8)

**Fig 5:**
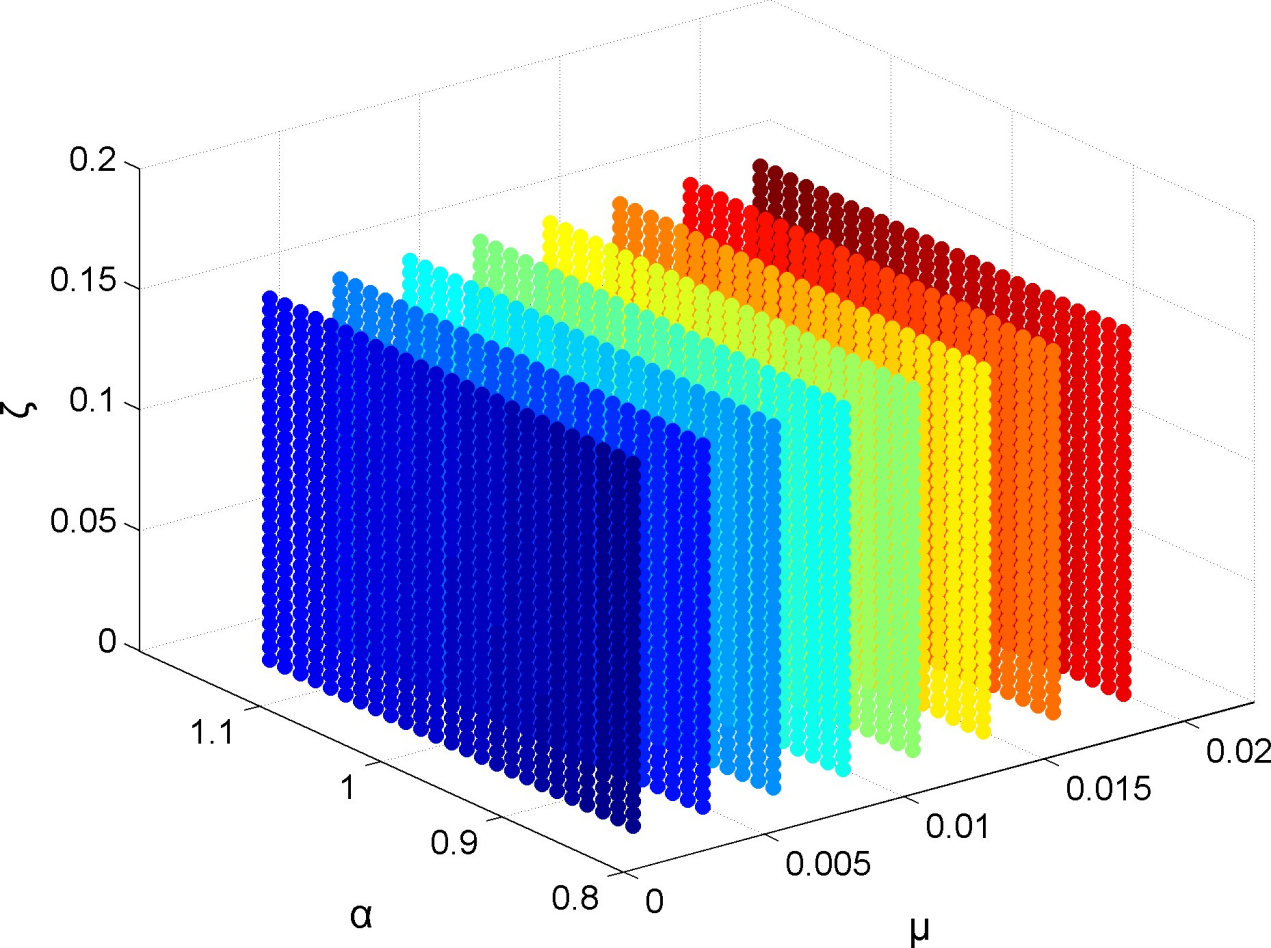
Ergodic search method solution space for TMD.

Presently, numerical optimization methods are used because the parameter optimization effect of analytic formula method on TMD is always limited, such as DH formula [11]. TMD numerical optimization methods commonly use a similar ergodic search method for calculations. In a TMD system, the ergodic search solution space shown in Fig 5 can be formed in the range of Eq (9). Search grid density is influenced by the value of each parameter in Eq (9). These can be set according to actual need. Ergodic search method grid accuracy greatly affects optimization.

$$\left\{ \begin{array}{l} \mu \in \left\{\hat{\mu }=\mu_{l}:\Delta \mu :\mu_{r}\right\}\\ \alpha \in \left\{\hat{\alpha }=\alpha_{l}:\Delta \alpha :\alpha_{r}\right\}\\ \zeta \in \left\{\hat{\zeta }=\zeta_{l}:\Delta \zeta :\zeta_{r}\right\} \end{array}\right.$$ (9)

Warburton [16] introduced a linear programming idea as a method for reducing the number of calculations required for an ergodic search [26]. An optimal solution for the linear programming problem presented in Fig 5 is usually obtained at the boundaries. A three-dimensional solution space boundary is equivalent to a cube’s six surfaces. If only boundary points are taken, calculation is greatly reduced. The number of calculations needed is reduced more when an ergodic search grid is more densely divided. Solving space is actually a domain of definition formed by discrete points, the method is called the "integer programming method". Warburton [16] and Wan [26] proved that under an appropriate boundary definition, an integer programming method can be used to obtain results which are close to the ergodic search method with less calculation.

Fan et al. used a traditional GA algorithm to study TMD parameters optimization on pedestrian-induced vibration and attempted to improve vibration reduction effects of a TMD system [14]. The specific implementation was: for the initial TMD parameters {*μ*_0_,*α*_0_,*ζ*_0_}, a new search limit was determined within a certain range in Eq (10). Optimal TMD parameters can be calculated via a GA search. However, for the parameters optimization of vehicle-bridge-TMD system, the GA algorithm still has the problem of low computational efficiency [27, 28].

$$\left\{ \begin{array}{l} \mu_{j}\in \left[\mu_{l},\mu_{r}\right]\\ \alpha_{j}\in \left[\alpha_{l},\alpha_{r}\right]\\ \zeta_{j}\in \left[\zeta_{l},\zeta_{r}\right] \end{array}\right.$$ (10)

## Variably accelerated pattern search algorithm based on Den Hartog formula

This paper proposes a variable, accelerated pattern search method (VAPS) which uses improved initial values. A Den Hartog (DH) formula is used to determine better initial search values and accelerate the convergence speed of traditional pattern search algorithm (PS). A traditional PS algorithm step length, and acceleration and deceleration, principles have been modified to improve search efficiency. Overall optimization is strengthened by improving initial values and traditional pattern search algorithm search processes. Partial search capabilities are improved and realize an optimal parameter solution for a TMD system.

The procedure is as follows:

1. A Den Hartog formula was preliminarily used to calculate the frequency ratio *α* and damping ratio *ζ* of the *n*-TMD system at different mass ratios *μ*, which was used as one of the initial values of the variably accelerated pattern search algorithm.
2. Using these search initial values, a VAPS algorithm performs vibration damping optimization calculations. Optimal *n*-TMD system parameters are selected according to the optimal displacement damping ratio.

The specific implementation is as follows.

### 4.1. Initial optimization preliminary determination using Den Hartog formula

The initial TMD parameters determinations can be performed using the DH formula with no main structure damping as the steel-box girder bridge actual damping ratio is very small. Guo and Lu proved that the DH formula was still applicable to TMD control of vehicle-bridge coupling vibration [10]. TMD systems with different mass ratios can be used to calculate design parameters using the DH formula. The results were used as one of the initial values (the DH initial value) in the follow-up procedure.

The value of *μ* is Eq (11). It shows that vector $\hat{\mu }$ is generated at equal intervals Δ*μ* within lower *μ*_*l*_, and upper, limits *μ*_*r*_. Parameter *μ* values are selected in the vector $\hat{\mu }$. The expression method in the following text is equivalent to Eq (11).

$$\mu \in \left\{\hat{\mu }=\mu_{l}:\Delta \mu :\mu_{r}\right\},\Delta \mu =0.005$$ (11)

### 4.2. VAPS algorithm accelerates convergence rate

**Fig 6:**
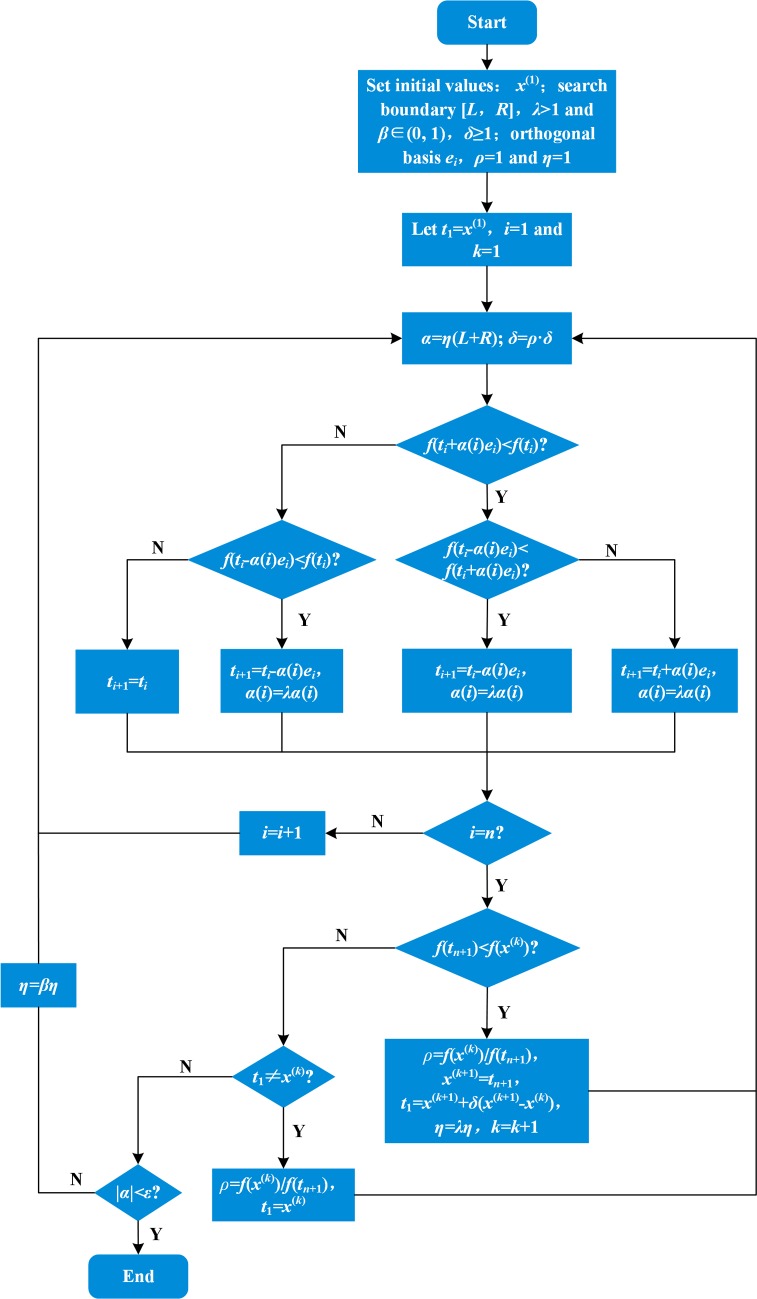
VAPS algorithm flow chart.

The pattern search algorithm is a special type of direct search algorithm [29], also known as the step acceleration method. This direct search algorithm uses the objective-function function value and does not calculate a derivative value. This is suitable for a number of nonlinear optimization problems present. A direct search algorithm usually has a slower convergence rate than a derivative method. A traditional pattern search (PS) algorithm and a variable step non-monotonic pattern search algorithm proposed by Yang, et al. [30], in a detection pattern iteration period, are relatively immobile. They are adjusted after a pattern movement has been obtained and result in a relatively large deviation between pattern movement direction and the optimal descent direction. It also restricts, to a degree, convergence speed and accuracy. Steps in each direction could be changed according to the actual situation by considering function value difference in each direction. Taking up the idea of simulated annealing [31], if a current search is distant from an optimal point, the acceleration factor can be enlarged. As a search approaches the optimal point, the acceleration factor may be reduced accordingly. VAPS algorithm steps and flow chart (Fig 6) are as follows.

Step 1:

Set TMD parameter optimization initial values as follows: 1) algorithm initial point *x*^(1)^; 2) initial step adjustment coefficient *η*; 3) search lower bound vector ***L***; and, 4) search upper bound vector ***R***. The initial step is ***α*** *= η*(***L***+***R***); let initial acceleration factor *δ*≥1, step increase coefficient *λ*>1, and step decrease coefficient *β*∈(0, 1). Acceleration factor *δ* adjustment factor is *ρ*. *e*_*i*_ is the orthogonal basis used of traditional pattern search method to specify the search direction [30]. The termination accuracy is *ε*. Let *t*_1_ = *x*^(1)^, *ρ* = 1, *η* = 1 and pattern move search loop step number *k* = 1.

Step 2:

Calculate the search step ***α*** *= η*(***L***+***R***).

Acceleration factor *δ* = *ρ*·*δ*, for *i* = 1, 2, …, *n*, the following judgment is made:

If *f*(*t*_*i*_+*α*(*i*)*e*_*i*_)<*f*(*t*_*i*_): if *f*(*t*_*i*_−*α*(*i*)*e*_*i*_)<*f*(*t*_*i*_+*α*(*i*)*e*_*i*_), then make *t*_*i*+1_ = *t*_*i*_−*α*(*i*)*e*_*i*_,*α*(*i*) = *λ*·*α*(*i*);

if *f*(*t*_*i*_−*α*(*i*)*e*_*i*_)≥*f*(*t*_*i*_+*α*(*i*)*e*_*i*_), then make *t*_*i*+1_ = *t*_*i*_+*α*(*i*)*e*_*i*_,*α*(*i*) = *λ*·*α*(*i*);

or, if *f*(*t*_*i*_−*α*(*i*)*e*_*i*_)<*f*(*t*_*i*_), then make *t*_*i*+1_ = *t*_*i*_−*α*(*i*)*e*_*i*_,*α*(*i*) = *λ*·*α*(*i*);

or, make *t*_*i*+1_ = *t*_*i*_.

Step 3:

If *f*(*t*_*n*+1_)<*f*(*x*^(*k*)^), then make *ρ* = *f*(*x*^(*k*)^)/*f*(*t*_*n*+1_), *x*^(*k*+1)^ = *t*_*n*+1_, *t*_1_ = *x*^(*k*+1)^+*δ*(*x*^(*k*+1)^−*x*^(*k*)^), *η* = *λ*·*η*; then make *k* = *k*+1, and go to Step 2.

Step 4:

If *t*_1_≠*x*^(*k*)^, then make *ρ* = *f*(*x*^(*k*)^)/*f*(*t*_*n*+1_), *t*_1_ = *x*^(*k*)^, and go to Step 2.

Step 5:

If |*α*(*i*)|<*ε*, then stop the calculation; or, make *η* = *β*·*η*, and go to Step 2.

The step adjustment coefficient *η* is no longer a number, but is an *n*×1 dimensional vector. At the beginning of the algorithm, initial step adjustment coefficients for all directions are equal. However, as detection movement progresses, the step adjustment coefficients in each direction varies according to function values in each direction. The *η* value returned either increase or decrease with structural maximum dynamic response results. After a cycle ends, it is then synchronically increased, or decreased, according to the overall situation. Similarly, the acceleration factor *δ* will increase, or decrease, as function value change after each mode movement. Therefore, a step in each direction probed by the VAPS algorithm can increase, or decrease, according to the function variation in each direction. This results in an algorithm mode movement direction closer to the optimal descent direction than an traditional PS algorithm. As the search progresses, the acceleration factor can be dynamically adjusted according to the objective function value change in the optimal descent direction. An optimum point is approached more quickly. When the search point closes to an optimum point, the acceleration factors decrease. This narrowing makes the searches for the final value of the algorithm more precise and facilitates finding the optimal point.

This VAPS algorithm allows an optimal *n*-TMD system parameter combination to be calculated according to the optimal displacement damping rate.

## Bridge overview and its dynamic analysis result

### 5.1. Steel-box girder bridge overview

**Fig 7:**
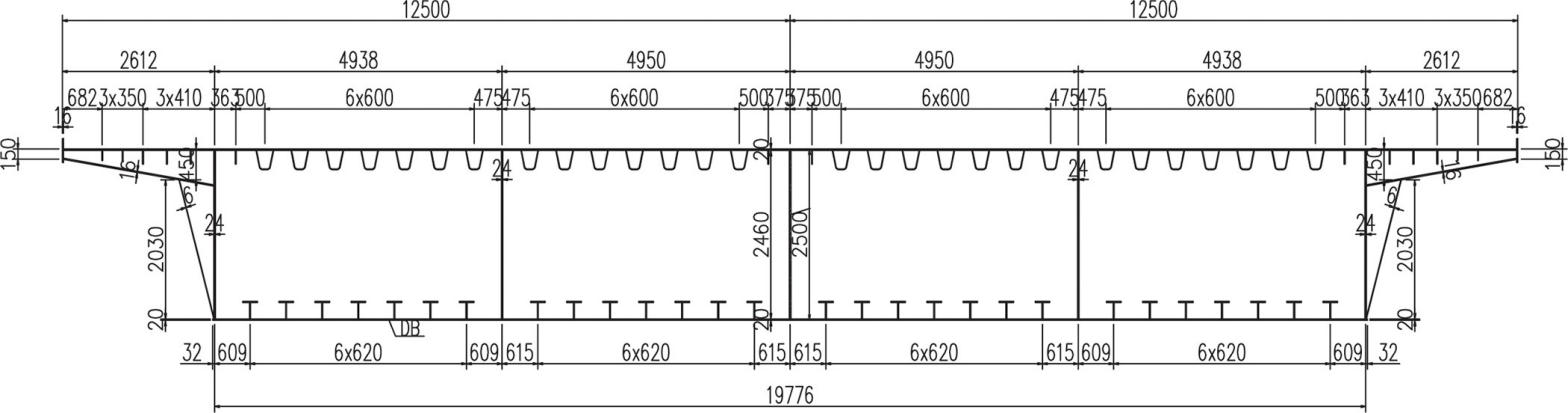
Standard cross section of steel-box girder.

Changsha's Hongqi viaduct bridge is used as an example. It is a continuous girder bridge with a uniform cross-section steel-box and a (42+64+42)m span. It has 12 lanes, 6 lanes in each direction. Its centerline curve radius is 600m. The main girder beam height is 2.5m. The top plate width is 25m. The cross-section is a 4-chamber box section of a whole single box (Fig 7).

An et al. [21] chose basic vehicle parameters which are shown in Table 1. When vehicle parameters or driving lanes differ, bridge vibrations caused by vehicles may offset one another. The TMD should control the most obvious vehicle-bridge vibration. This paper used vehicle fleet loading and passage along the same lane parameters as Miguel, L. F. F., et al [19]. When vehicles cross the bridge at equal time intervals longitudinally, power amplification becomes apparent. A 10-vehicle motorcade travelling longitudinally at 20 meter intervals was chosen. The vehicles were assumed to drive precisely in the preceding vehicle's path.

**Table 1:** Vehicle parameters.

Parameter	Unit	Value	Parameter	Unit	Value
*m*_*c*_	kg	20000	*d*_1_	m	1.5
*J*_*xc*_	kg·m^2^	55160	*d*_2_	m	2.5
*J*_*yc*_	kg·m^2^	13490	*b*_1_, *b*_2_	m	1.1
*m*_*wa*_, *m*_*wb*_	kg	710	*m*_*wc*_, *m*_*wd*_	kg	800
*k*_1*a*_, *k*_1*b*_	N/m	3.99×10^5^	*k*_1*c*_, *k*_1*d*_	N/m	3.99×10^6^
*c*_1*a*_, *c*_1*b*_	N·s/m	2.32×10^4^	*c*_1*c*_, *c*_*1d*_	N·s/m	2.32×10^4^
*k*_2*a*_, *k*_2*b*_	N/m	3.51×10^5^	*k*_2*c*_, *k*_2*d*_	N/m	3.51×10^5^
*c*_2*a*_, *c*_2*b*_	N·s/m	800	*c*_2*c*_, *c*_2*d*_	N·s/m	800

### 5.2. Natural vibration characteristics analysis

**Fig 8:**
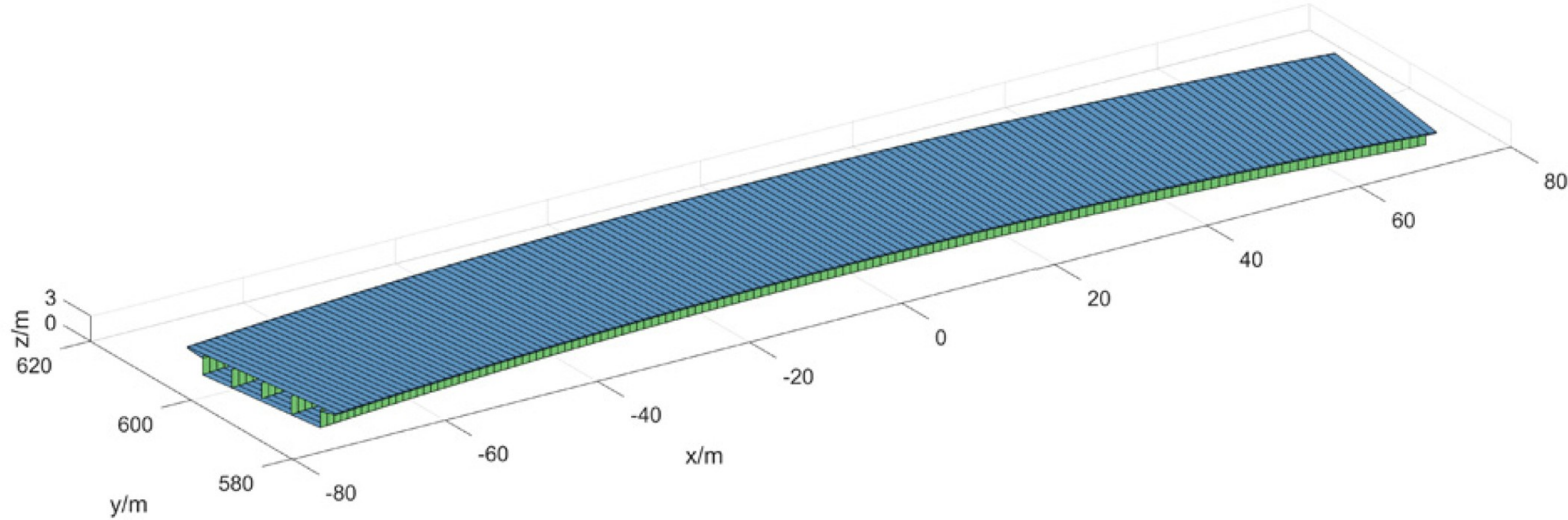
7-DOF beam element model built by VBTS-1.

**Fig 9:**
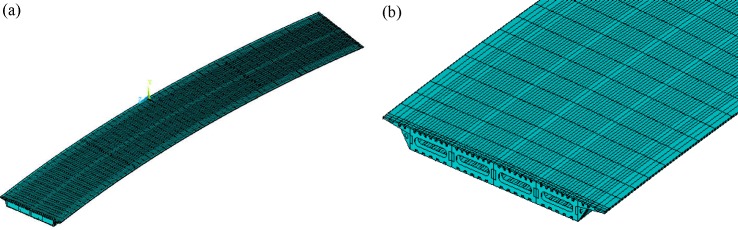
ANSYS detailed model of steel-box girder bridge. (a) This is full bridge FEM model. (b) This is box girder end FEM model.

VBTS-1 self-programming software established a curved beam element model (Fig 8). Longitudinal stiffening ribs were included in top and bottom board thickness. Diaphragm plate torsional stiffness was included in top and bottom boards. The bridge model has a total of 148 units. A refined finite element model was formed using ANSYS software Shell 181 shell elements (Fig 9). The ANSYS model has a total of 357,574 units. Longitudinal and transverse stiffening ribs, bearing stiffening ribs and piers, diaphragm plates and other parts were simulated. A modal analysis was performed using a Lanczos method [32]. The first five orders of bridge natural frequencies and mode shapes were chosen in Table 2. The calculation results of the two models are essentially consistent which tends to verify the reliability of the self-programming software.

**Table 2:** Bridge natural vibration characteristics comparison.

Mode number	VBTS-1 frequency (Hz)	ANSYS frequency (Hz)	Mode characteristic
1	2.665	2.650	1st order symmetric vertical bending
2	5.120	5.106	1st order anti-symmetric vertical bending
3	5.325	5.331	2nd order symmetric vertical bending
4	5.993	5.977	1st order torsion
5	7.822	7.971	2nd order torsion

### 5.3. Resonant vehicle speed and controlled modes

The dynamic impact factor (IM), which is the measure of vehicle-bridge coupled vibration effect, is calculated as follows [4]:

$$\mathrm{IM}=\left(R_{\mathrm{dyn}}-R_{\mathrm{sta}}\right)/R_{\mathrm{sta}}$$ (12)

where *R*_dyn_ and *R*_sta_ = bridge maximum dynamic and static responses, respectively.

**Fig 10:**
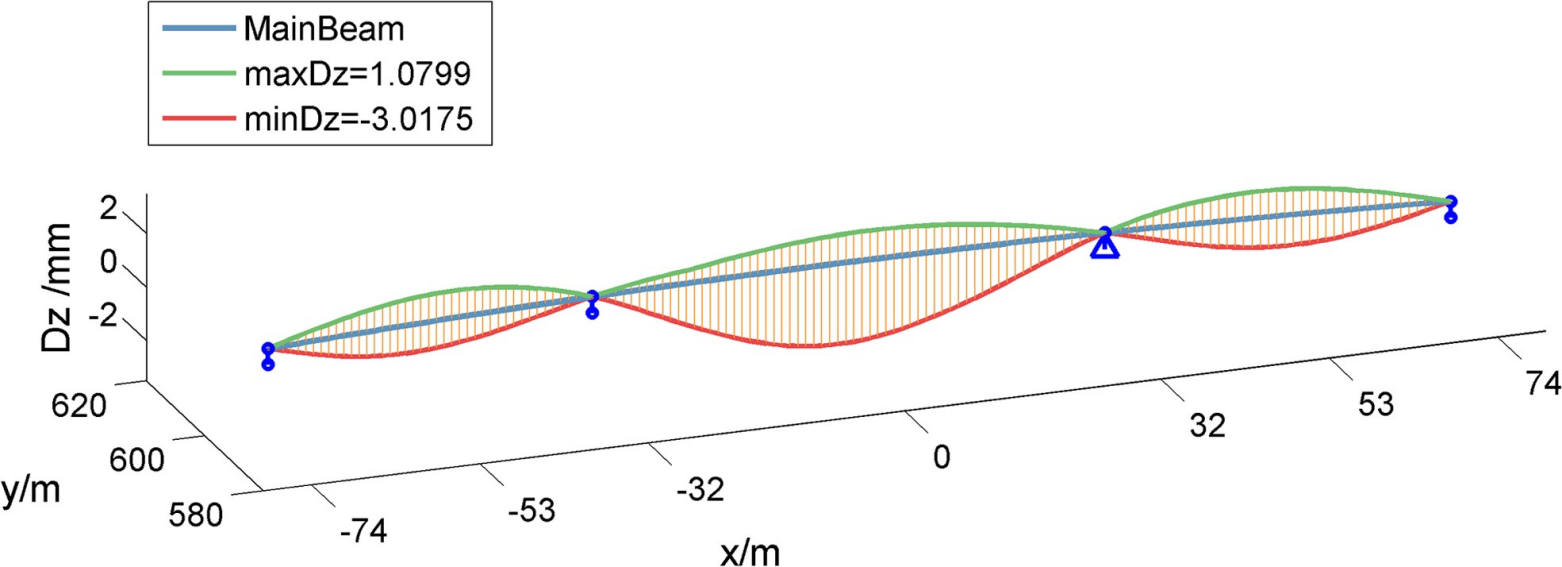
Static structural displacement envelope diagram under vehicle load.

**Fig 11:**
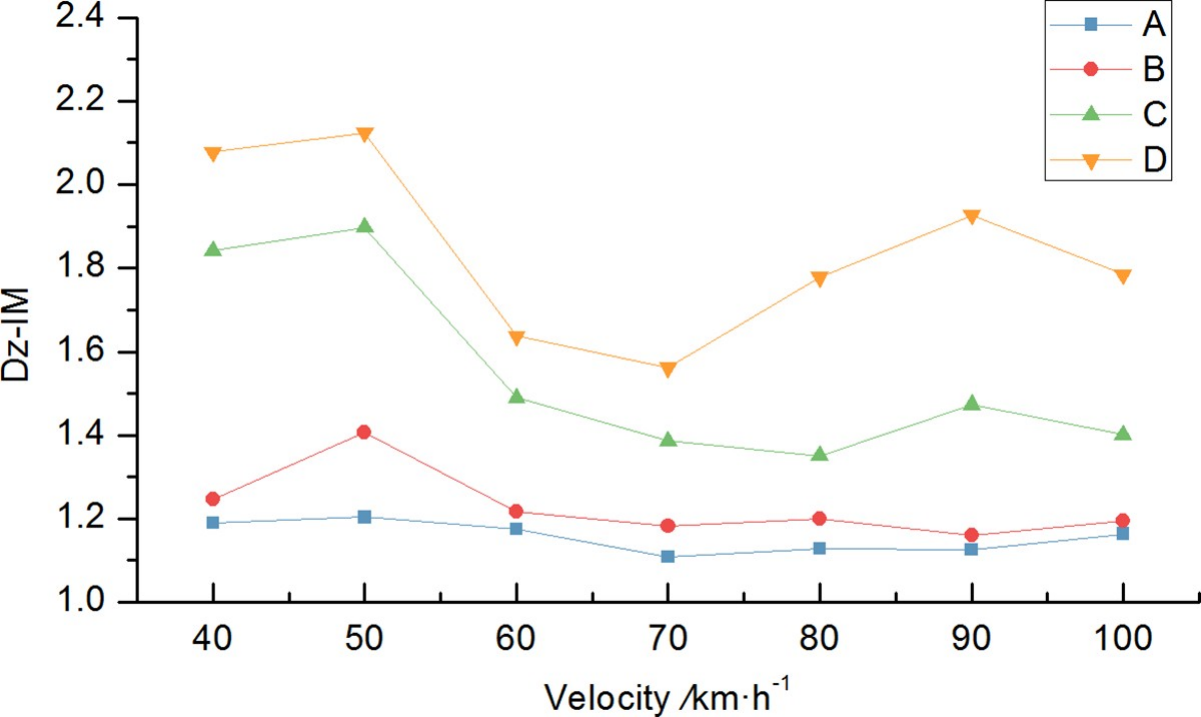
Midspan displacement IM under varying road surface conditions.

The Chinese national standard road with four common road surface conditions: A (very good); B (good); C (acceptable); and D (poor) were analyzed. The static displacement and the dynamic IM of the bridge midspan displacement under the vehicle load were calculated assuming that the single motorcade drove along the outermost lane with equal spacing and constant speed (Figs 10 and 11). This is an unfavorable condition. The results show that bridge dynamic responses fluctuates under varying conditions with the vehicle speed. There is a resonance phenomenon, which is when vehicle speed is close to resonance speed, at vehicle speeds of 50km/h and 90km/h. The bridge dynamic response has a significant amplification effect.

**Fig 12:**
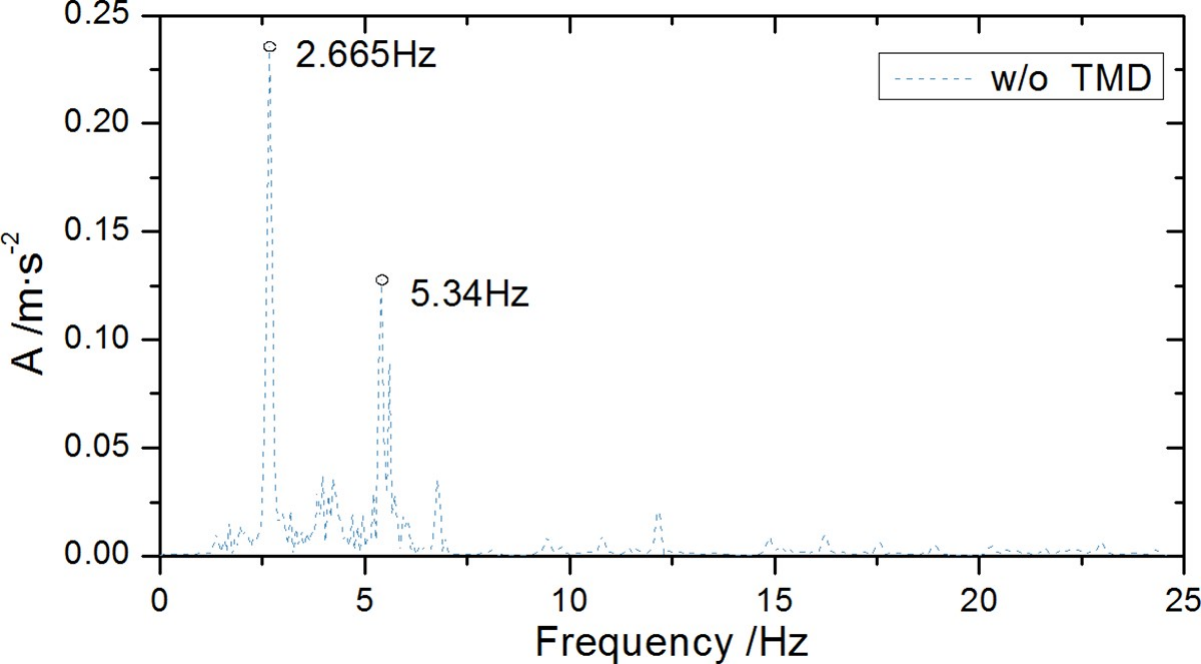
Midspan acceleration spectrums without TMD.

Resonance speed is 50km/h under C-level road condition. Bridge midspan spectrum curve response is calculated (Fig 12). Structural vibration frequency distribution characteristic analysis shows that bridge vibration mode is primarily first-order vertical bending. The corresponding natural vibration frequency is 2.665Hz, which was selected as controlled vibration mode.

## Steel-box girder bridge TMD parameter optimization

This section discusses different TMD parameter optimization methods, considering both displacement response damping effect and optimization calculations time costs. It compares and evaluates various methods to obtain the most suitable TMD damping optimization method for a three-span curve continuous steel-box girder bridge.

### 6.1. Single variable optimization based on Den Hartog formula

**Fig 13:**
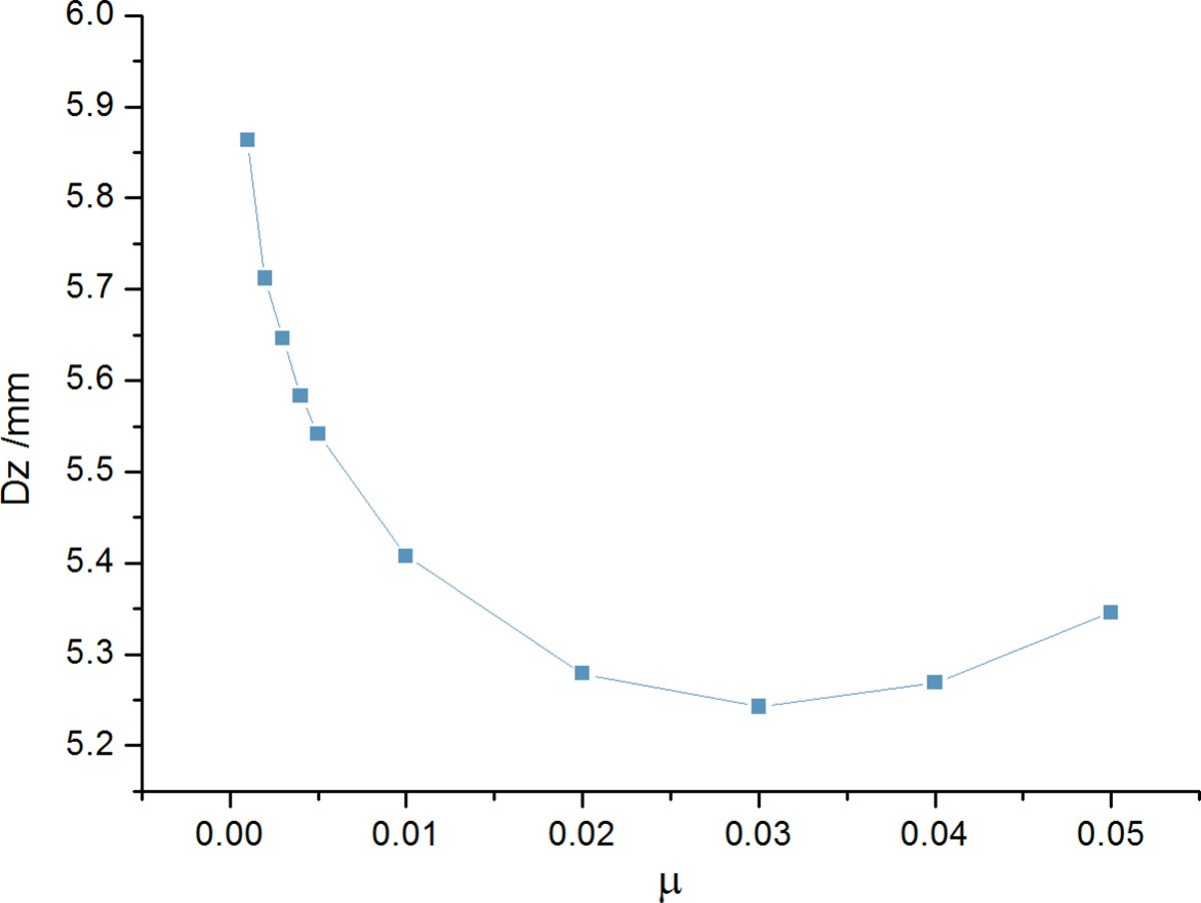
Midspan dynamic response under different mass ratios.

A single TMD system was placed at the bridge midspan cross-section for the resonant vehicle speeds and controlled mode. The mass ratio *μ* range was 0.001 to 0.05. The optimum frequency ratio *α*_*opt*_ and damping ratio *ζ*_*opt*_ were determined using DH optimization formula. The maximum displacement dynamic response of the bridge midspan under various mass ratios were calculated (Fig 13). Displacement dynamic response obtains its minimum value when mass ratio is 0.03 (mass *m* = 17950kg). The corresponding 1-TMD initial optimization parameter is: {*μ* = 0.03,*α* = 0.9709,*ζ* = 0.1015}.

**Fig 14:**
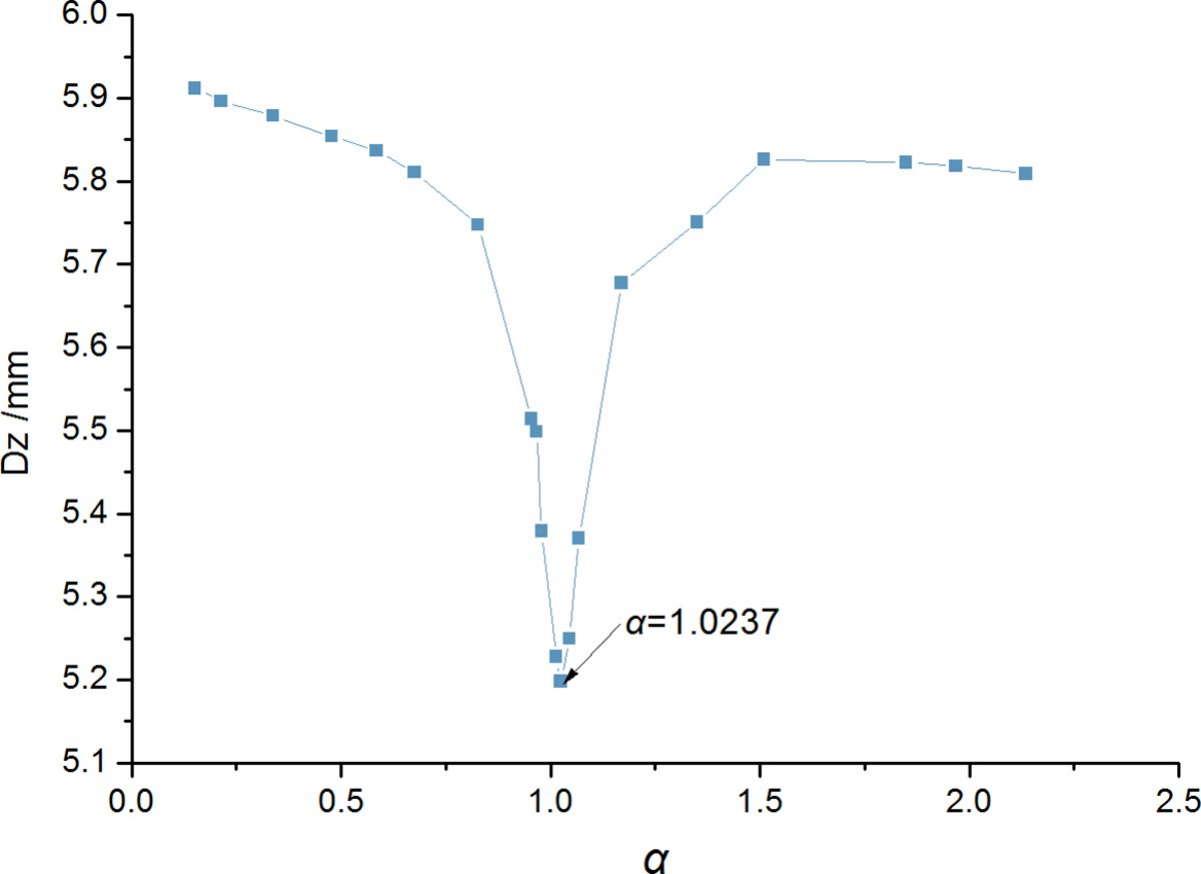
Midspan dynamic response under different frequency ratios.

The frequency ratio range was 0.2 to 2.2. Mass ratio and damping ratio were unchanged. Bridge midspan response values were calculated (Fig 14). When the frequency ratio was 1.0237 (stiffness factor of *K* = 5285kN/m), response maximum value across the middle node was minimized. The 1-TMD parameter value was {*μ* = 0.03,*α* = 1.0237,*ζ* = 0.1015}.

**Fig 15:**
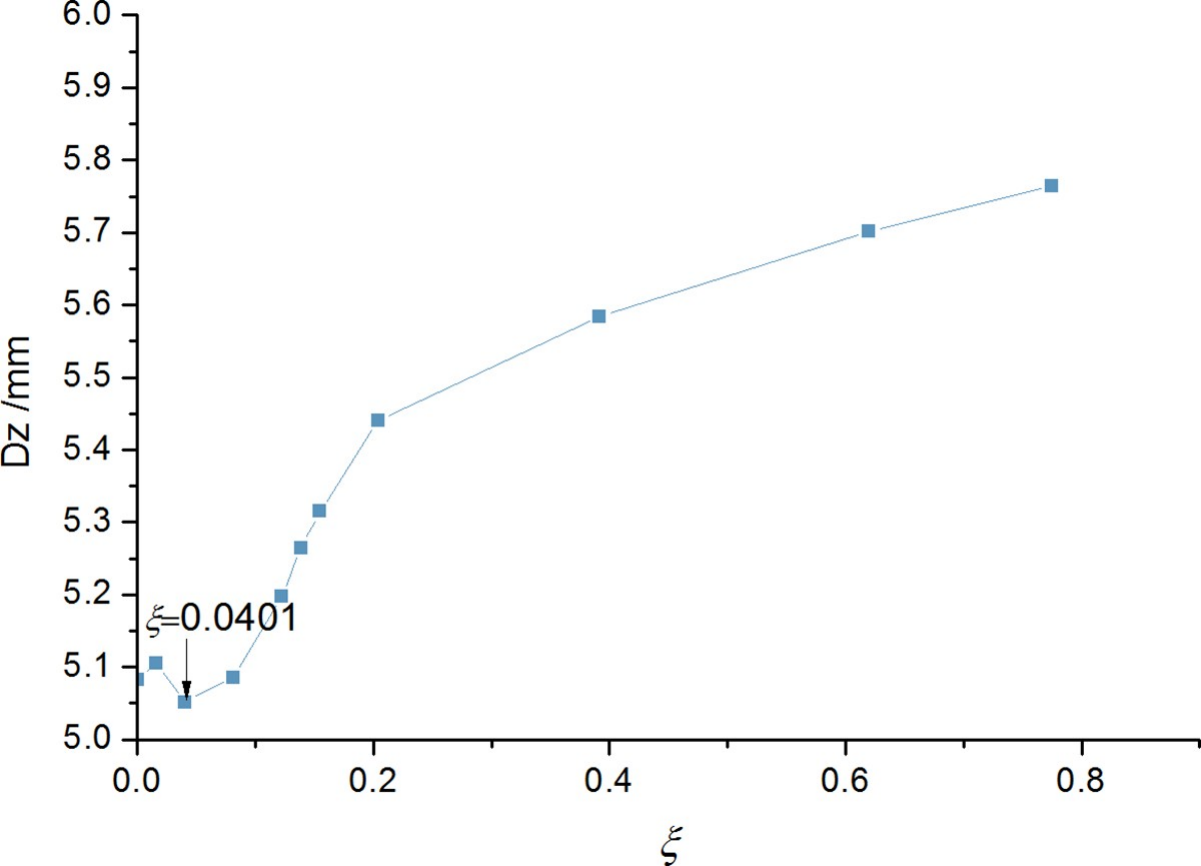
Midspan dynamic response under different damping ratios.

The damping ratio range was 0.0 to 0.8. Mass ratio and frequency ratio remained constant. Bridge midspan response values were calculated (Fig 15). The curve trend was analyzed. Damping ratio was 0.0401 with a damping coefficient of *C* = 24.62kNs/m, when the damping effect was optimal. A 3-TMD system divided equally in weight was placed in the midspan cross-section and symmetrically arranged along the cross-bridge at equal intervals, in order to avoid any adverse effect of a single TMD mass which might be too much heavy for the structure. Each TMD value is: {*μ* = 0.0100, *α* = 1.0237, *ζ* = 0.0134}. The maximum displacement of the bridge midspan were calculated as 5.052 mm and 0.677 m·s^-2^ respectively, whose vibration damping rates were 28.5% and 32.6%, respectively.

### 6.2. Multivariate optimization based on ergodic search method

**Fig 16:**
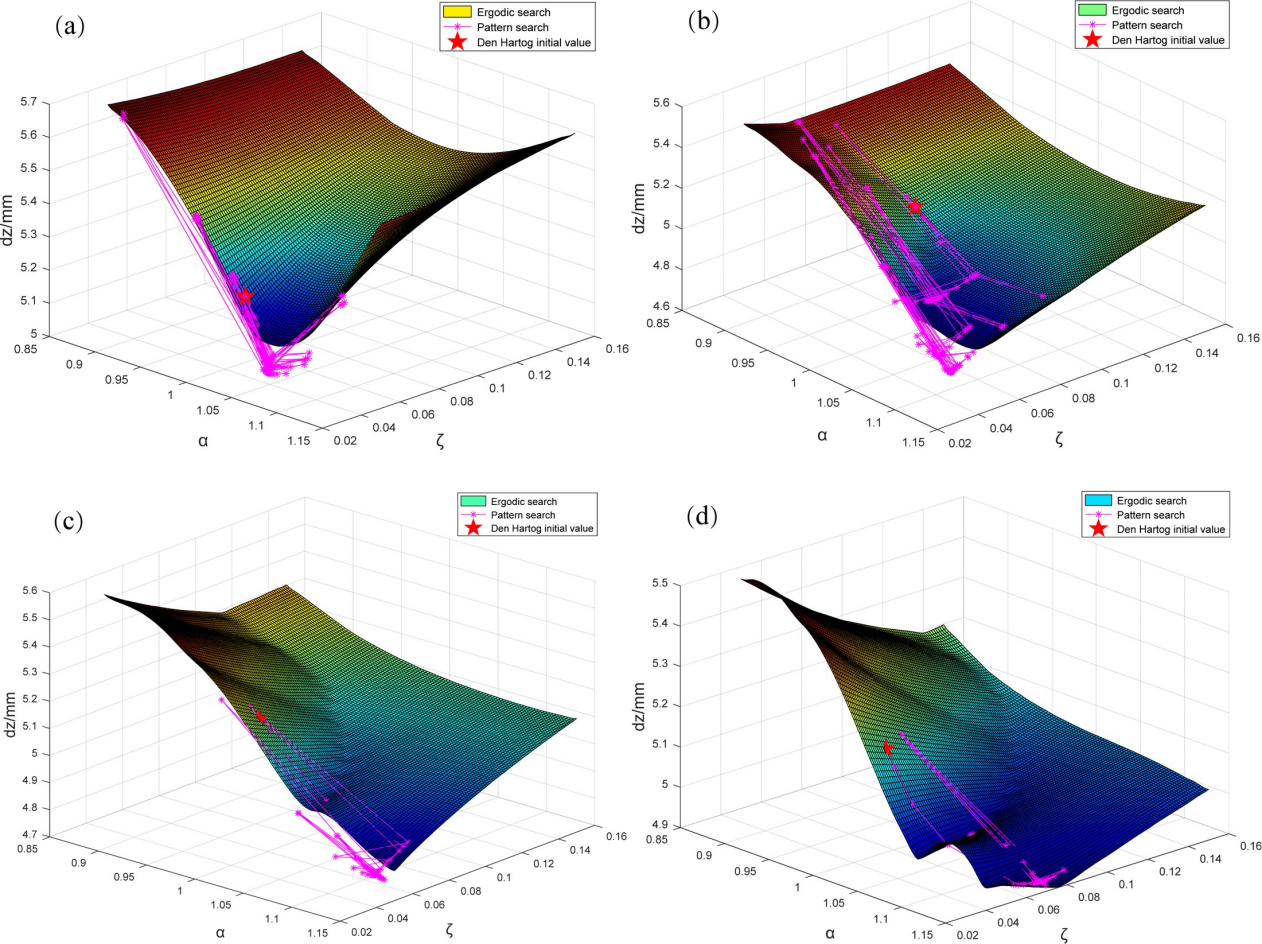
Displacement response comparison between ergodic search and traditional PS when *μ* remains constant. (a) *μ* remains 0.005. (b) *μ* remains 0.010. (c) *μ* remains 0.015. (d) *μ* remains 0.020. Taking Fig 16(A) as an example, while keeping TMD parameter *μ* = 0.005 unchanged, the results obtained by the ergodic search method for different *α* and *ζ* are fitted to the space surface in the graph after Matlab surface fitting. The coordinate values of the three directions of each point on the curve surface represent the values of *α* and *ζ*, and the corresponding midspan displacement amplitude. Similarly, for the traditional PS algorithm, the “*”point on the purple curve in the figure depicts the change process of *α* and *ζ* in each search and the corresponding midspan displacement amplitude, and the five-pointed star indicates the pattern search initial point calculated based on DH formula. It can be seen that the pattern search method can quickly converge to the optimal solution from an ideal initial point after a certain search process, thereby greatly reducing the number of searches for TMD optimization calculation.

An ergodic search was performed within the range of Eq (13). The maximum mid-bridge span dynamic displacement response under partial *μ* values was extracted. Using MATLAB [33], the surface fitting is plotted (Fig 16). The results were compared with the traditional PS algorithm optimization results using DH initial value. *μ* value was unchanged during the optimization process.

The analysis shows that: 1) under different TMD parameters combinations, structural maximum displacement responses vary greatly. It has no consistent change trend and shows a strong irregularity. In Fig 16(D), there are two "bottoms" showing there are two optimal solutions under this assumption; 2) The optimal solution under the same *μ* value often appears near the 3D graph boundary. An integer programming method can be used to calculate the power response corresponding to the parameter combination located on the ergodic search space boundary and an acceptable combination of parameters can be obtained more quickly; and, 3) The traditional PS algorithm, under the initial value of DH results, is consistent with the ergodic search method, and quickly approached the "bottom" with short calculation times. The best vibration damping solution is better than the ergodic search. Specific optimum parameters and damping effects are in Section 6.6.

$$\left\{ \begin{array}{l} \mu \in \left\{\hat{\mu }=0.0025:0.0025:0.02\right\}\\ \alpha \in \left\{\hat{\alpha }=0.85:0.025:1.15\right\}\\ \zeta \in \left\{\hat{\zeta }=0.01:0.01:0.15\right\} \end{array}\right.$$ (13)

### 6.3. Multivariable optimization based on integer programming method

**Fig 17:**
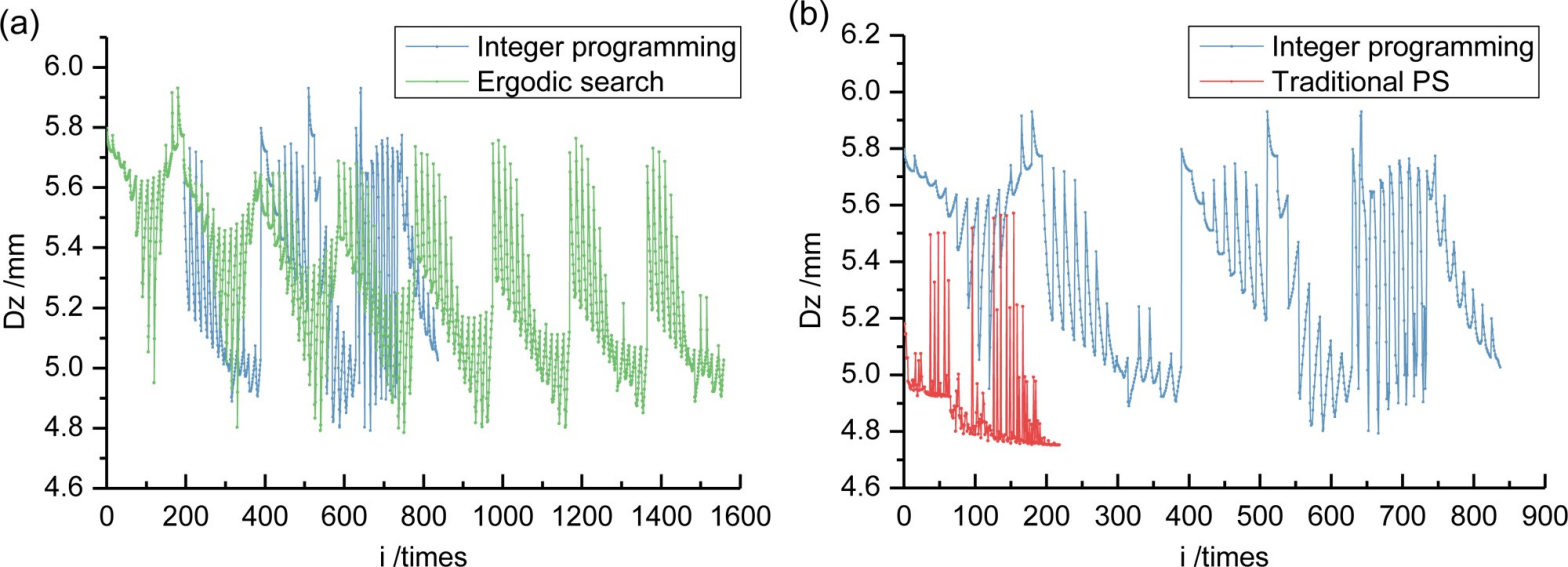
Optimization process comparison of displacement response among integer programming, ergodic search, and traditional PS (using DH initial value when *μ* = 0.01). (a) This is a comparison between integer programming and ergodic search. (b) This is a comparison between integer programming and traditional PS.

Section 6.2 gave the results for an ergodic search solution space formed by Eq (13). The {*μ*, *α*, *ζ*} parameter combination corresponding to the points on the six boundary faces are selected to calculate the bridge dynamic response after installing a TMD. The specific optimum parameters and damping effects are presented in the following Section 6.4. The calculation number is selected as the abscissa. The ordinate of the corresponding dynamic response result, is plotted in Fig 17. This was done in order to further reflect the optimization effects of different methods. It can be seen that: 1) Using the integer programming method, results close to the ergodic search method can be obtained in shorter calculation times; 2) The traditional PS algorithm, under the initial DH value had the fewest calculations and the best optimization effects. It is a suitable TMD vibration damping optimization algorithm.

### 6.4. Multivariate optimization based on traditional genetic algorithm

**Fig 18:**
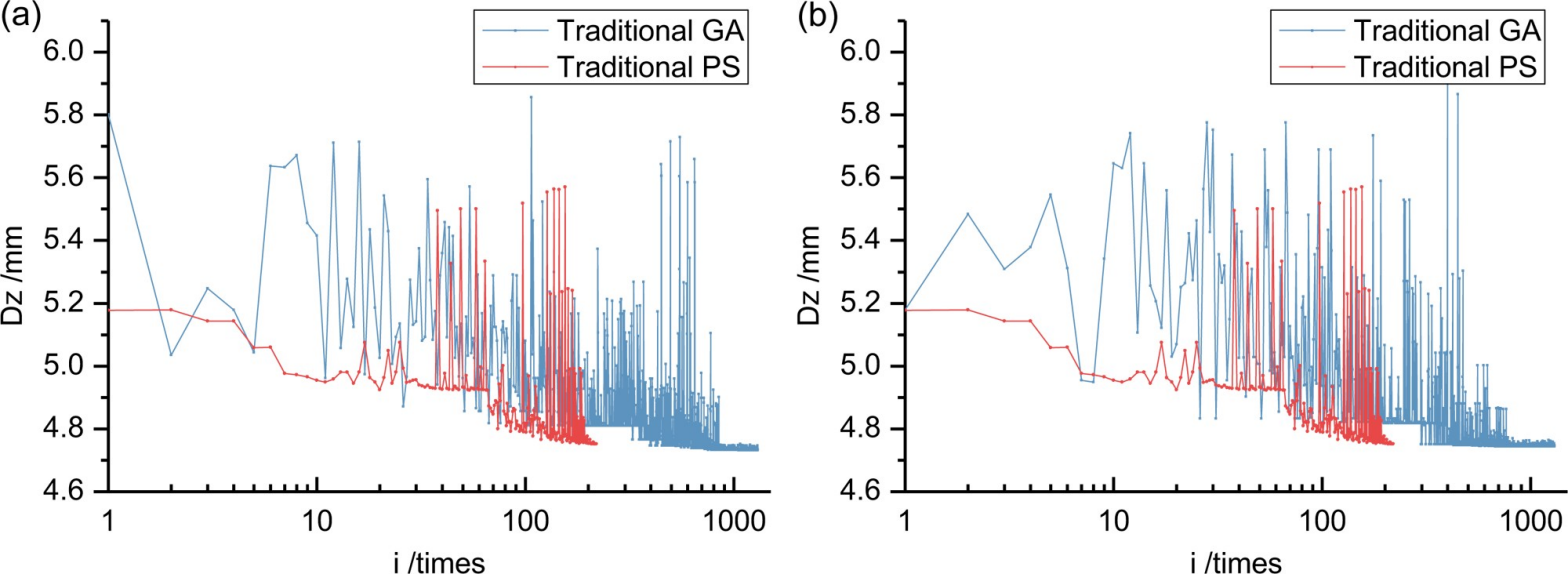
Optimization process comparison of dynamic response between traditional GA and PS (using DH initial value when *μ* = 0.01). (a) This is first GA algorithm calculation process. (b) This is second GA algorithm calculation process.

**Fig 19:**
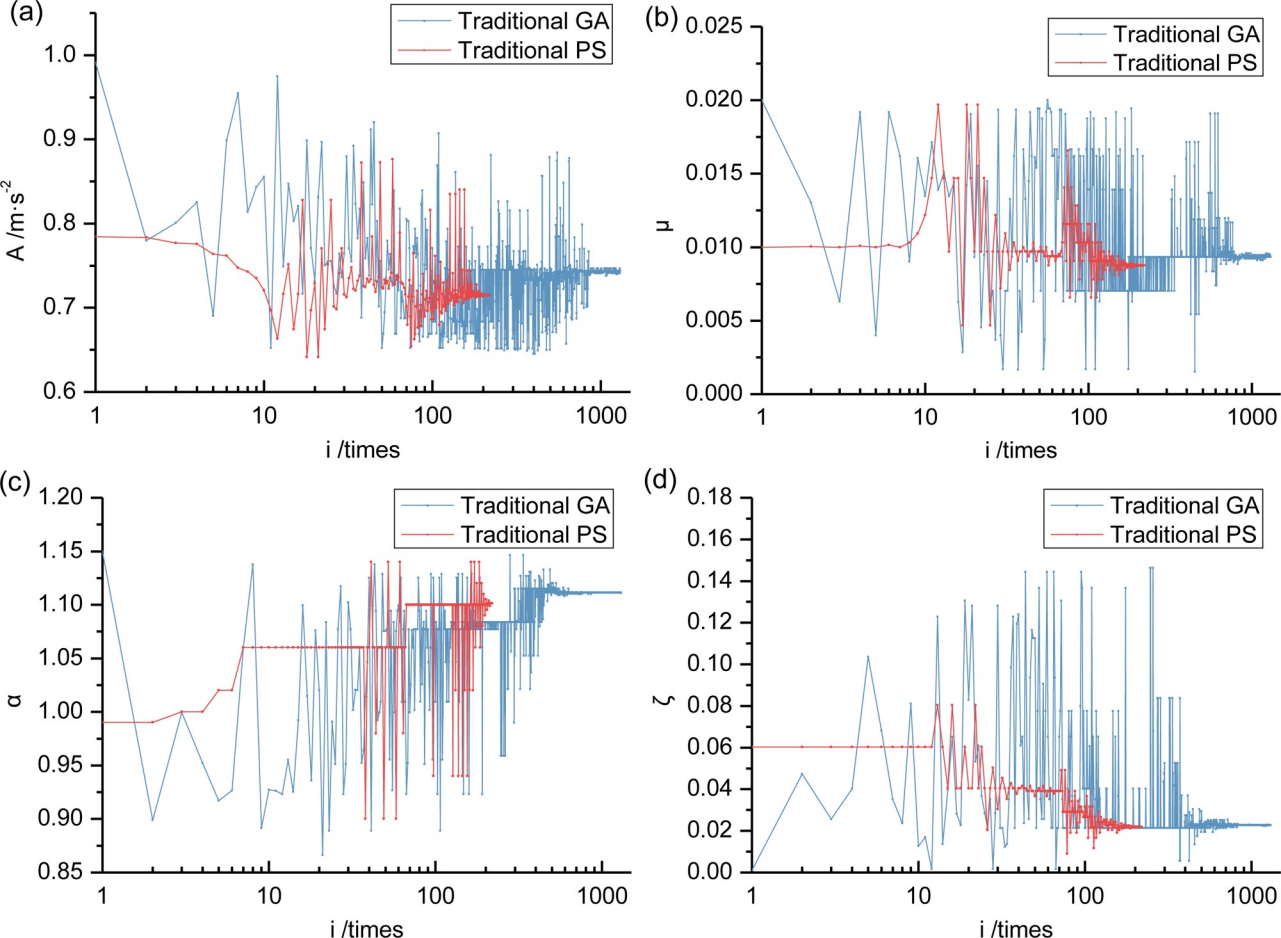
Optimization process comparison of other parameters between second traditional GA and PS (using DH initial value when *μ* = 0.01). (a) This is about acceleration *A*. (b) This is about mass ratio *μ*. (c) This is about frequency ratio *α*. (d) This is about damping ratio *ζ*.

This section compares the GA algorithm and the PS algorithm to confirm the applicability to solve the TMD vibration optimization problem. A "roulette" selection operator in the GA algorithm results in differing optimization processes [34]. The following compares the GA algorithm optimization process and the PS algorithm (Figs 18 and 19). The GA algorithm has lengthy calculation times, so a logarithmic axis is used in the abscissa. Specific optimum parameters and damping effects are given in Section 6.6.

The analysis shows that: 1) the {*μ*, *α*, *ζ*} parameter of the traditional PS algorithm search range is smaller than the traditional GA algorithm. Its overall search ability is not as good as the latter but it quickly converges under superior initial optimization values. Optimization results quite close to the latter are obtained with relatively few calculations and reflect the superiority of the traditional PS algorithm in the TMD damping optimization analysis; 2) Under the two optimization algorithms, the final values of {*μ*, *α*, *ζ*} are quite similar, which tends to confirm the reliability of the optimization results.

### 6.5. Multivariable optimization based on variably accelerated pattern search algorithm

**Fig 20:**
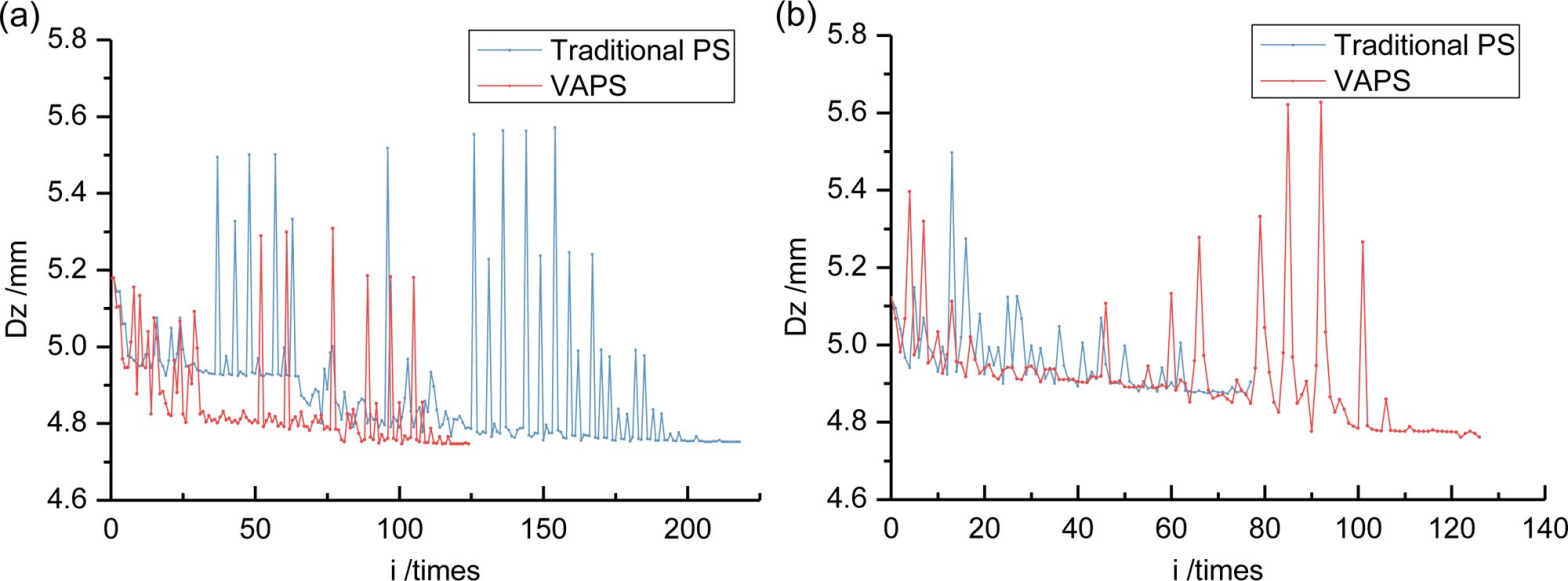
Optimization process comparison of dynamic response between traditional PS and VAPS using DH initial value. (a) This is a comparison when *μ* = 0.01. (b) This is a comparison when *μ* = 0.02.

**Fig 21:**
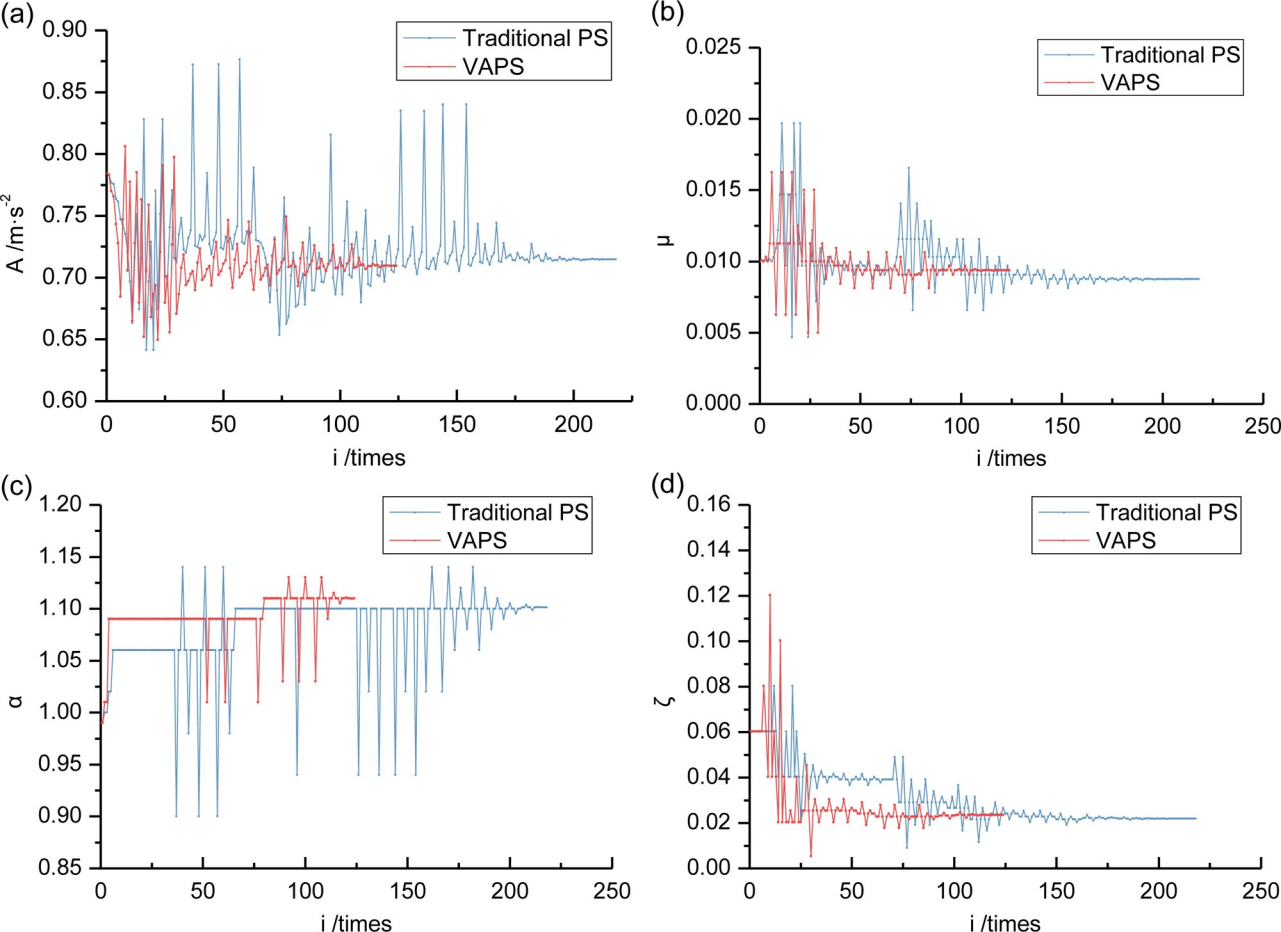
Optimization process comparison of other parameters between traditional PS and VAPS using DH initial value when *μ* = 0.01. (a) This is about acceleration *A*. (b) This is about mass ratio *μ*. (c) This is about frequency ratio *α*. (d) This is about damping ratio *ζ*.

Traditional PS algorithm with a DH formula already provide effective TMD vibration-damping optimization methods. In an attempt to further improve TMD optimization calculations efficiency, this section discusses the optimization effects between the traditional PS algorithm and the VAPS algorithm in DH initial values corresponding to different *μ* values. Results are compared (Figs 20 and 21). Specific optimum parameters and damping effects provided in Section 6.6.

The results show that: 1) the two PS algorithm both converge quickly under different initial values and their convergent results are close. This indicates that the dependence of PS method on initial value has been improved under the DH formula; 2) The VAPS algorithm achieves superior optimization results with fewer calculations using *μ* = 0.01 as the initial DH value, which suggests a significant variably-accelerated improvement in traditional PS algorithm. TMD-damping optimization calculation convergence rates are accelerated; and, 3) With an initial DH value when μ = 0.02, a VAPS algorithm has a greater number of calculations compared to a traditional PS algorithm. But it achieves superior damping optimization results with higher optimization guarantees compared to a traditional PS algorithm, whose premature convergence causes a less effective optimization. 4) Final {*μ*, *α*, *ζ*} values, returned by the two optimization algorithms are quite close, the difference <5%, which tends to confirm optimization reliability.

### 6.6. Different methods optimization effects compared

The displacement damping rate for the TMD system, *β*, is defined as:

$$\beta =1-\left(D_{z}-D_{0}\right)/\left(D_{\max}-D_{0}\right)$$ (14)

*D*_*z*_ is the absolute value of the maximum displacement at the corresponding bridge node installed with the TMD system; *D*_*0*_ is the absolute value of the maximum static displacement without TMD system; *D*_max_ is the absolute value of the maximum dynamic displacement without TMD system.

The optimal parameter combination of 3-TMD obtained via different optimization methods and the corresponding optimization calculation times are extracted. The vibration-damping effect is calculated according to Eq (14) and summarized in Table 3.

**Table 3:** 3-TMD optimized results compared.

Optimization method	*μ*_*opt*_	*α*_*opt*_	*ζ*_*opt*_	*D*_*z*_(mm)	*A*(m/s2)	Calculation times	Displacement damping ratio
DH-based method	0.0100	1.0237	0.0134	5.052	0.677	——	28.5%
Ergodic search	0.0100	1.1250	0.0200	4.785	0.701	1560	37.9%
Integer programming	0.0100	1.1000	0.0100	4.792	0.718	838	37.7%
Traditional GA	0.0093	1.1113	0.0227	4.744	0.710	1300	39.4%
Traditional PS	0.0088	1.1013	0.0219	4.752	0.715	219	39.1%
VAPS	0.0094	1.1101	0.0234	4.747	0.710	125	39.2%

The results show:

1. The traditional GA algorithm, the traditional PS algorithm, and the VAPS algorithm have superior optimization results when measured by displacement damping rate. A Den Hartog formula-based, single-variable optimization method has the worst effect.
2. The VAPS algorithm has the fewest calculations and the most efficient vibration damping optimization calculation rate. When compared to traditional PS algorithm, a VAPS algorithm can often reduce time by half. The other methods' optimization efficiency is deficient by comparison. Traditional GA algorithm and ergodic search methods could take 10 times as long.
3. Results can be achieved through an integer programing method with fewer calculations and are close to ergodic search methods. The number of calculations can be reduced more when densely partitioning the ergodic search grid.
4. The optimized final {*μ*, *α*, *ζ*} values obtained by the traditional GA algorithm, the traditional PS algorithm and the VAPS algorithm are quite close and tend to confirm optimization results reliability.

The VAPS algorithm under an initial DH value requires relatively few calculations. It can accelerate TMD damping optimization calculation convergence rates in the premise of superior vibration damping effect. It can also search for parameter combinations of {*μ*, *α*, *ζ*} over a large range with certain overall search capabilities.

## Comparison of dynamic response results with and without TMD

**Fig 22:**
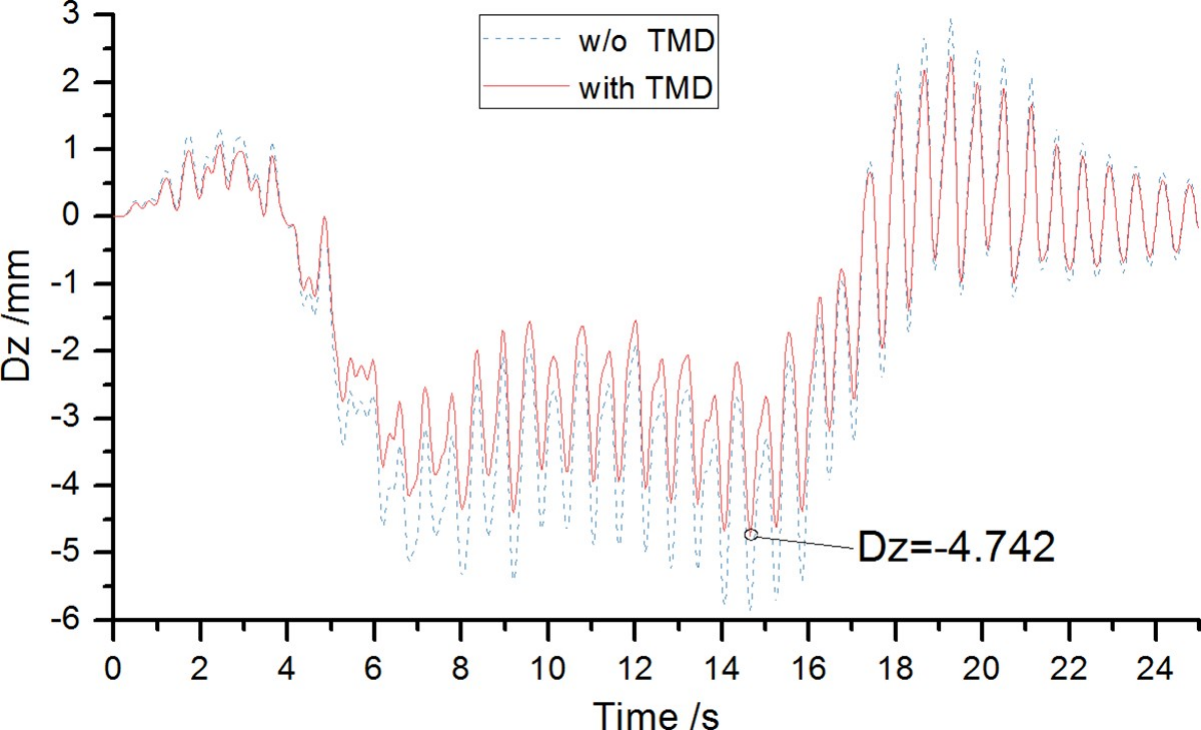
Midspan displacement time-history curves with and without TMD.

Comparing the operating conditions before, and after installing, the TMD system, the optimal 3-TMD displacement damping effects are calculated and shown in Fig 22. By analyzing time-displacement curves, it can be seen intuitively that displacement dynamic response after the TMD installation is weakened. Comparing the different stages of the displacement time history curves before and after TMD system installation, it can be seen that there are significant differences: 1) When the motorcade initially enters the bridge, the difference in displacement response between the presence and absence of the TMD system is very small, due to the TMD damping activation delay. In the first few vibration cycles, there is almost no vibration damping effect; 2) As the motorcade is on the bridge, i.e. the 8~16s part of the time history curve, there is a stable vibration phase, and bridge displacement response maximizes. TMD system has significant vibration damping effects; 3) After the motorcade exits the bridge, the bridge freely vibrates. After a TMD system installation, amplitude decreases and decay speeds up.

**Fig 23:**
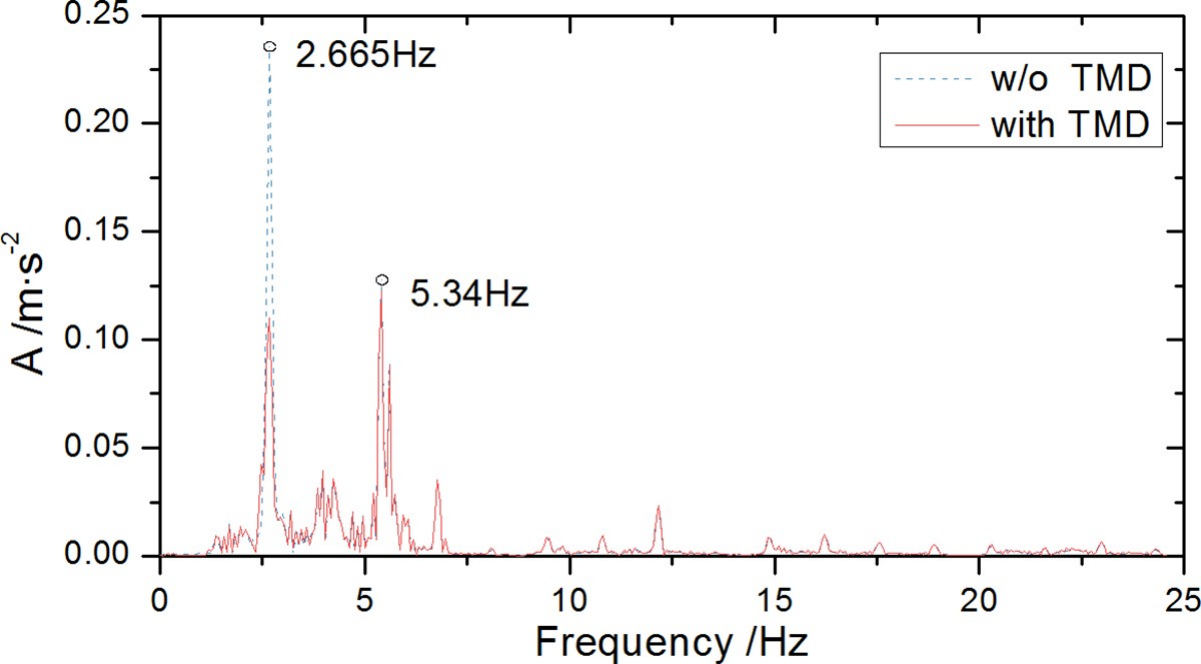
Midspan acceleration spectrums with and without TMD.

The frequency response result of the acceleration time-history response dispersed by Fourier transform is shown in Fig 23 showing the control effects of the controlled frequency after the TMD system installation. The analysis shows that: after the TMD damping device installation, first-order frequency peak amplitude of the bridge acceleration spectrum is greatly attenuated, indicating that it suppresses the contribution of the peak frequency to structural vibration. The vibration control effect on the controlled mode (*f* = 2.665Hz) is achieved, thereby effectively reducing the bridge dynamic responses.

## Conclusions

For the optimization problem of vehicle-bridge coupling damping for steel box-girder bridges, a dynamic analysis model of vehicle-bridge-TMD coupled system was established. A variably accelerated pattern search algorithm based on a Den Hartog formula was proposed. The VBTS-1 software with a visual interface, programmed in Fortran language, was used for an optimization study of vibration damping. A three-span curved continuous steel box girder bridge situated on Hongqi Road, Changsha was taken the example. A discussion on the parameter optimization of TMD system was performed. Their optimized effects under different methods were compared. The comparison included a single variable optimization method based on a Den Hartog formula, an ergodic search method, an integer programming method, a traditional genetic algorithm, a traditional pattern search algorithm, and a variably accelerated pattern search algorithm. Finally, the vibration reduction effects before and after the optimized TMD system installation were compared. The main conclusions are as follows:

1. Comprehensively considering displacement response damping effects and the optimization calculations time cost, a VAPS algorithm under Den Hartog initial values has relatively fewer calculation times and the optimization effect seem certain compared to various TMD parameter optimization methods. A single-variable optimization method based on a Den Hartog formula has the least calculation number but the least effective damping effect. A traditional GA algorithm has the best damping effect, but the longest calculation time.
2. Final TMD parameter optimization values obtained by different methods are quite close to each other and tend to verify the reliability of the optimization results. This paper has positive significance for the variably accelerated improvement on a traditional PS algorithms. It can speed up TMD damping optimization calculation convergence rate under a premise of ensuring the optimization effect.
3. The optimal parameter combination of a 3-TMD system obtained by a VAPS algorithm based on Den Hartog initial value has a displacement damping ratio of 39.2%, an increases of 10.7% compared to a single variable optimization based on a Den Hartog formula.
4. Large-scale unconstrained optimization for MTMD systems become feasible via VAPS algorithm computational efficiency improvements due to optimizing calculation time which exponentially increases with the number of its TMD components.

## Supporting information

S1 Appendix Detailed descriptions on variables in this paper. (DOCX)
