# Supplementary material for: Study on optimization algorithm of tuned mass damper parameters to reduce vehicle-bridge coupled vibration
Source: PLoS One. 2019 Apr 23;14(4):e0215773. doi: 10.1371/journal.pone.0215773 (PMC6478324; doi:10.1371/journal.pone.0215773)
Supplement: S1 Appendix — (DOCX) [file pone.0215773.s001.docx]

Appendix A

Detailed descriptions on variables in this paper are as follows.

**Table A1. Detailed descriptions on variables in this paper.**

| **Variable** | **Significance of variable** | **Significance of subscript and superscript** |
| --- | --- | --- |
| $\mathbf{M}_{s},\mathbf{M}_{v},\mathbf{M}_{t}$ | overall mass matrix; | *s* means structure; *v* means vehicle; *t* means TMD |
| $\mathbf{C}_{s},\mathbf{C}_{v},\mathbf{C}_{t}$ | overall damping matrix; |  |
| $\mathbf{K}_{s},\mathbf{K}_{v},\mathbf{K}_{t}$ | overall stiffness matrix; |  |
| $\mathbf{V}_{s},\mathbf{V}_{v},\mathbf{V}_{t}$ | column vector of displacement in the overall coordinates |  |
| $z_{c},z_{wa},z_{wb},z_{wc},z_{wd}$ | vertical displacement of car body and wheels; | *c* means car body; *w* means wheel; *a*/*b*/*c*/*d* respectively means wheels' number |
| $m_{c},m_{wa},m_{wb},m_{wc},m_{wd}$ | mass of car body and wheels |  |
| $z'_{a},z'_{b},z'_{c},z'_{d},$ | wheel's vertical displacement at the junction of axle and wheel; | *s* means structure; *w* means wheel; *a*/*b*/*c*/*d* respectively means wheels' number |
| $z_{swa},z_{swb},z_{swc},z_{swd}$ | bridge's vertical displacement at the the junction of bridge deck and wheel |  |
| $\theta_{yc},\theta_{xc}$ | rotational displacement about corresponding coordinate axis | *x*/*y* means coordinate axis; *c* means car body |
| $k_{1a},k_{1b},k_{1c},k_{1d}$ | stiffness factor of vehicle model | 1 means 1st spring-damping system of car body; 2 means 2nd spring-damping system of car body; *a*/*b*/*c*/*d* respectively means wheels' number |
| $k_{2a},k_{2b},k_{2c},k_{2d}$ |  |  |
| $c_{1a},c_{1b},c_{1c},c_{1d}$ | damping factor of vehicle model |  |
| $c_{2a},c_{2b},c_{2c},c_{2d}$ |  |  |
| $J_{xc},J_{yc}$ | rotational inertia about corresponding coordinate axis | *x*/*y* means coordinate axis; *c* means car body |
| $b$ | car body width; | 1 means left; 2 means right |
| $b_{1}\text{,}b_{2}$ | distance between the left and right wheels and mass center of car body |  |
| $d$ | distance between the front and rear axles; | 1 means front; 2 means rear |
| $d_{1}\text{,}d_{2}$ | distance between the left and right wheels and mass center of car body |  |
| $k_{tk}$ | stiffness factor of TMD | *t* means TMD; *k* means the order of TMD |
| $c_{tk}$ | damping factor of TMD; |  |
| $m_{tk}$ | mass of TMD; |  |
| $v_{tk}$ | displacement of TMD |  |
| $\mathbf{C}_{sf}^{v},\mathbf{C}_{sf}^{t},\mathbf{C}_{vf}$ | additional damping matrix; | Subscript: *s* means structure; *v* means vehicle; *t* means TMD; *f* means append; *o* means coupling.  Superscript: *v* means the source is vehicle; *t* means the source is TMD |
| $\mathbf{C}_{svo},\mathbf{C}_{vso},\mathbf{C}_{sto},\mathbf{C}_{tso}$ | coupled damping matrix; |  |
| $\mathbf{K}_{sf}^{v},\mathbf{K}_{sf}^{t},\mathbf{K}_{vf}$ | additional stiffness matrix; |  |
| $\mathbf{K}_{svo},\mathbf{K}_{vso},\mathbf{K}_{sto},\mathbf{K}_{tso}$ | coupled stiffness matrix; |  |
| $\mathbf{p}_{svcr},\mathbf{p}_{svkr}$ | force vector of vehicle on bridge caused by road irregularity; | *s* means structure; *v* means vehicle; *c* means damping force; *k* means elastic force; *r* means road roughness |
| $\mathbf{p}_{vvcr},\mathbf{p}_{vvkr}$ | force vector of bridge on vehicle caused by road irregularity; |  |
